# Sodium fluctuation as a parameter in predicting mortality in general hospitalized patients

**DOI:** 10.3389/fmed.2024.1399638

**Published:** 2024-07-16

**Authors:** Siyu Liang, Lize Sun, Yuelun Zhang, Qi Zhang, Nan Jiang, Huijuan Zhu, Shi Chen, Hui Pan

**Affiliations:** ^1^Department of Endocrinology, Key Laboratory of Endocrinology of National Health Commission, Translation Medicine Center, Peking Union Medical College Hospital, Peking Union Medical College, Chinese Academy of Medical Sciences, Beijing, China; ^2^Eight-year Program of Clinical Medicine, Peking Union Medical College Hospital, Peking Union Medical College, Chinese Academy of Medical Sciences, Beijing, China; ^3^Central Research Laboratory, Peking Union Medical College Hospital, Peking Union Medical College, Chinese Academy of Medical Sciences, Beijing, China; ^4^Department of Clinical Laboratory, Peking Union Medical College Hospital, Peking Union Medical College, Chinese Academy of Medical Sciences, Beijing, China; ^5^4+4 Medical Doctor Program, Peking Union Medical College Hospital, Peking Union Medical College, Chinese Academy of Medical Sciences, Beijing, China

**Keywords:** dysnatremia, sodium fluctuation level, prediction, single parameter system, mortality

## Abstract

**Background:**

Dysnatremia is the most common electrolyte disorder in hospitalized patients. Sodium fluctuation level may be a better parameter in dysnatremia management. We aimed to examine the association between sodium fluctuation level during hospitalization and mortality and to evaluate its value in predicting poor prognosis among general hospitalized patients.

**Methods:**

Data were collected from patients admitted to Peking Union Medical College Hospital. The generalized estimated equation (GEE) was used to examine the relationship between sodium fluctuation level and mortality. Receiver-operating characteristic (ROC) curve analysis was performed to calculate the optimal cutoff value and the area under the ROC curve (AUC).

**Results:**

Sodium fluctuation level showed a dose-dependent association with increased mortality in general hospitalized patients. After adjusting age, sex, length of hospital stay, and Charlson comorbidity index, the ORs of group G2 to G6 were 5.92 (95% CI 5.16–6.79), 26.45 (95% CI 22.68–30.86), 50.71 (95% CI 41.78–61.55), 104.38 (95% CI 81.57–133.58), and 157.64 (95% CI 112.83–220.24), respectively, *p* trend <0.001. Both normonatremia and dysnatremia patients on admission had the dose-dependent associations similar to general hospitalized patients. The AUC of sodium fluctuation level was 0.868 (95% CI 0.859–0.877) in general hospitalized patients, with an optimal cutoff point of 7.5 mmol/L, a sensitivity of 76.5% and a specificity of 84.2%.

**Conclusion:**

We determined that sodium fluctuation level had a dose-dependent association with increased mortality in general hospitalized patients. Sodium fluctuation level could be used to develop a single parameter system in predicting mortality in general hospitalized patients with acceptable accuracy, sensitivity, and specificity.

## 1 Introduction

Dysnatremia is the most common electrolyte disorder in hospitalized patients. Dysnatremia is classified into hyponatremia and hypernatremia. Hyponatremia is defined as a serum sodium level below 135 mmol/L and is observed in 14–22% hospitalized patients (1, 2). Hypernatremia is regarded as a serum sodium level above 145 mmol/L and is found in 21–26% hospitalized patients (3, 4). Previous studies have shown that both hyponatremia and hypernatremia were independently associated with poor prognosis (3, 5). Severe hyponatremia may cause cerebral edema (6). While hypernatremia contributes to cerebral dehydration, resulting in epileptic seizures, coma, or respiratory arrest (7). Therefore, accurate identification of high-risk dysnatremia patients is crucial for precise management.

In recent years, several studies noted that hyponatremia and hypernatremia frequently occurred in the same patient within a short period; this condition was defined as mixed dysnatremia (8). Patients with mixed dysnatremia were associated with an increased risk of mortality (9), suggesting that sodium fluctuation level during hospitalization could be more accurate in reflecting disease severity than serum sodium level. In addition, patients with serum sodium levels between reference ranges (135 mmol/L-145 mmol/L) are regarded as low-risk patients. However, recent studies suggested that sodium fluctuations were associated with mortality even within sodium reference ranges (10). Therefore, considering the clinical significance of mixed dysnatremia and sodium fluctuations within the normal range, sodium fluctuation level during hospitalization may be a better parameter in dysnatremia management.

The poor prognostic impact of sodium fluctuation during hospitalization has been evaluated in patients with normonatremia on admission (11–13). However, these studies ignored patients with dysnatremia on admission. Patients admitted with dysnatremia frequently combined with chronic hyponatremia or hypernatremia. Thus, when these patients experience additional sodium fluctuations during hospitalization, the management of dysnatremia will be more difficult, leading to an increased risk of mortality. We hypothesized that sodium fluctuation during hospitalization was also associated with poor prognosis in patients with dysnatremia on admission. Therefore, this study included patients with normonatremia and dysnatremia on admission to evaluate the value of sodium fluctuation level during hospitalization in predicting mortality in general hospitalized patients.

The aim of our study is (i) to determine the association between sodium fluctuation level during hospitalization and the risk of mortality; (ii) to evaluate the value of sodium fluctuation level in predicting prognosis among general hospitalized patients.

## 2 Materials and methods

### 2.1 Study design

The single-center retrospective cohort study was conducted at Peking Union Medical College Hospital (Beijing, China). Patients admitted between 1 January 2015 and 9 August 2020 were included in this study. Both data collection and follow-up were from the Electronic Medical Record (EMR), and the data collection stopped after the patients were discharged.

The study was approved by the Ethics Committee of Peking Union Medical College Hospital, Chinese Academy of Medical Sciences (approval number: S-k1272, approval date: 9 October 2020). This study was reported according to The Strengthening the Reporting of Observational Studies in Epidemiology (STROBE) (14).

### 2.2 Participants

Participants were from Peking Union Medical College Hospital, Chinese Academy of Medical Sciences. Patients were included if they (i) were over 18 years of age; (ii) had at least two serum sodium measurements during hospitalization. Patients were excluded if they (i) had incomplete diagnostic codes; (ii) were without serum sodium measurement within 24 h after admission; or (iii) had conflict serum sodium levels in the same laboratory test. The inclusion and exclusion of participants were based on the records of EMR.

### 2.3 Study variables

All variables were collected through EMR, including age, sex, diagnosis codes, laboratory tests, and intensive care unit (ICU) transfer. General hospitalized patients were classified into 2 groups according to their serum sodium level on admission: (i) normonatremia on admission (serum sodium level between 135 mmol/L and 145 mmol/L on admission); (ii) dysnatremia on admission (serum sodium level below 135 mmol/L or above 145 mmol/L on admission). The minimum serum sodium level was defined as the lowest record of serum sodium measurements during a single hospitalization. The maximum serum sodium level was defined as the highest record of serum sodium measurements during a single hospitalization. Sodium fluctuation level was defined as difference between the maximum and minimum serum sodium levels during a single hospitalization.

According to previous studies, sodium fluctuation level below 6.0 mmol/L was treated as normal and was associated with a relatively low risk of death (10). Therefore, we divided patients into 6 groups to examine the dose-dependent relationship between sodium fluctuation level and mortality: G1 group (sodium fluctuation level < 6.0 mmol/L), G2 group (sodium fluctuation level between 6.0 mmol/L and 12.0 mmol/L), G3 group (sodium fluctuation level between 12.0 mmol/L and 18.0 mmol/L), G4 group (sodium fluctuation level between 18.0 mmol/L and 24.0 mmol/L), G5 group (sodium fluctuation level between 24.0 mmol/L and 30.0 mmol/L), and G6 group (sodium fluctuation level > 30.0 mmol/L).

We further categorized the primary diagnoses according to the International Classification of Diseases, 10th Revision, and calculated the Charlson comorbidity index (CCI) to measure comorbidity (15). The diagnostic codes were shown in Supplementary Table S1.

### 2.4 Outcomes

Our primary outcome was mortality, which was extracted from EMR. Several patients discharged against medical advice (AMA) had poor prognoses. However, they chose to leave the hospital due to various reasons (16). Considering the high mortality rate in AMA discharged patients in this study center, we defined mortality as in-hospital mortality and AMA discharged.

### 2.5 Statistical analysis

Categorical variables were reported as count (%), and continuous variables were reported as mean with standard deviation (SD). The Cochran-Armitage test was used to assess trends between categorical variables.

The primary analysis was the GEE. We chose GEE because our study comprised 20,164 re-hospitalized patients. In our analysis, the sodium fluctuation level was defined as the difference between the maximum and minimum serum sodium levels observed during a single hospitalization. Consequently, the readmitted patients had multiple measurements of sodium fluctuation levels. Prior studies have also utilized GEE to analyze repeated measurements (17, 18). Hence, we opted for GEE to model the relationship between sodium fluctuations, the number of hospitalizations, and mortality, and to test the main effect of sodium fluctuation on mortality. The GEE approach identified the repeated measurements by patient ID, and an exchangeable matrix was used as the working matrix. The odds ratio (OR) with 95% confidence interval (CI) were calculated to examine (i) the association and overall trends between sodium fluctuation level and mortality; and (ii) the association between sodium fluctuation level during hospitalization, minimum serum sodium level, maximum serum sodium level and mortality. The variance inflation factor test was performed to avoid the collinearity of the variables included in the models. Sensitivity analysis was further used to adjust the impact of AMA discharge on patients’ outcomes.

We used receiver-operating characteristic (ROC) curve analysis to investigate the ability of a single laboratory value, such as sodium fluctuation level during hospitalization, minimum serum sodium level, or maximum serum sodium level to predict mortality. The patient-specific predicted survival values and optimal cutoff value were derived from the ROC curve analysis. AUC was calculated based on original value of sodium fluctuation level during hospitalization, minimum serum sodium level, or maximum serum sodium level. The Delong test was used to compare the difference between AUCs of sodium fluctuation level during hospitalization, minimum serum sodium level, and maximum serum sodium level. The Brier score and calibration metrics were derived from logistic regression model.

All analyses were conducted with R (version 4.0.2, R Foundation for Statistical Computing, Vienna, Austria, 2020^1^).

## 3 Results

### 3.1 Demographic and characters

**Figure 1:**
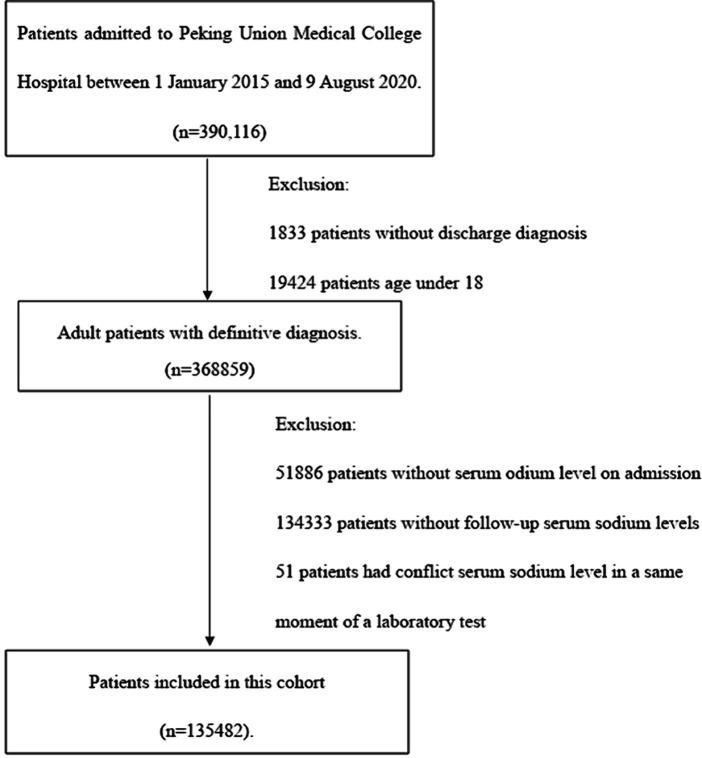
Flow chart of inclusion and exclusion of study cohort.

A total of 390,116 patients admitted to Peking Union Medical College Hospital between 1 January 2015 and 9 August 2020 were screened for inclusion. After excluding non-eligible cases, 135,482 patients were included in this study, as shown in Figure 1. The baseline characters of patients were summarized in Table 1. Patients had a mean age of 54.15 years (SD 15.95), with a mean CCI of 2.11 (SD 2.52). The cohort had a similar number of men and women, and 70,037 (51.7%) of 135,482 patients were female. The average of hospital stays was 13.24 days (SD 13.26) in general hospitalized patients, 12.77 days (SD 12.21) in normonatremia patients on admission, and 18.33 days (SD 20.95) in dysnatremia patients on admission. 1912 (1.4%) patients died in hospital or had an AMA discharge.

**Table 1:** Baseline characters and outcomes of normonatremia patients on admission and dysnatremia patients on admission.

		General hospitalized patients	Normonatremia patients on admission	Dysnatremia patients on admission
Demographic				
*N*, %		135,482	124,056	11,426
Age, years		54.15 (15.95)	53.82 (15.82)	57.69 (16.94)
Age, *n* (%)				
	18–65 years	100,795 (74.4)	93,391 (75.3)	7,404 (64.8)
	66–75 years	23,416 (17.3)	21,077 (17.0)	2,339 (20.5)
	> 75 years	11,271 (8.3)	9,588 (7.7)	1,683 (14.7)
Sex, *n* (%)				
	Female	70,037 (51.7)	64,855 (52.3)	5,182 (45.4)
	Male	65,445 (48.3)	59,201 (47.7)	6,244 (54.6)
Comorbidities				
Myocardial infarction, *n* (%)		2,675 (2.0)	2,311 (1.9)	364 (3.2)
Congestive heart failure, *n* (%)		10,591 (7.8)	9,149 (7.4)	1,442 (12.6)
Peripheral vascular disease, *n* (%)		13,874 (10.2)	12,597 (10.2)	1,277 (11.2)
Cerebrovascular disease, *n* (%)		11,069 (8.2)	9,794 (7.9)	1,275 (11.2)
Dementia, *n* (%)		351 (0.3)	282 (0.2)	69 (0.6)
Chronic pulmonary disease, *n* (%)		7,686 (5.7)	6,781 (5.5)	905 (7.9)
Connective tissue disease, *n* (%)		8,324 (6.1)	7,165 (5.8)	1,159 (10.1)
Ulcer disease, *n* (%)		2,544 (1.9)	2,145 (1.7)	399 (3.5)
Diabetes				
	Without end organ damage, *n* (%)	18,127 (13.4)	16,059 (12.9)	2,068 (18.1)
Hemiplegia	With end organ damage, *n* (%)	998 (0.7)	894 (0.7)	104 (0.9)
		383 (0.3)	325 (0.3)	58 (0.5)
Tumor				
	Tumor without metastasis, *n* (%)	35,919 (26.5)	33,248 (26.8)	2,671 (23.4)
	Metastatic solid tumor, *n* (%)	13,411 (9.9)	11,805 (9.5)	1,606 (14.1)
Leukemia, *n* (%)		1,202 (0.9)	1,048 (0.8)	154 (1.3)
Lymphoma, *n* (%)		3,624 (2.7)	2,907 (2.3)	717 (6.3)
Liver disease				
	Mild liver disease, *n* (%)	14,476 (10.7)	13,150 (10.6)	1,326 (11.6)
	Moderate or severe liver disease, *n* (%)	1,505 (1.1)	1,162 (0.9)	343 (3.0)
Moderate or severe renal disease, *n* (%)		13,397 (9.9)	11,140 (9.0)	2,257 (19.8)
Acquired immune deficiency syndrome, *n* (%)		107 (0.1)	84 (0.1)	23 (0.2)
Charlson Comorbidities Index		2.11 (2.52)	2.04 (2.48)	2.89 (2.86)
Average length of hospital stays, day		13.24 (13.26)	12.77 (12.21)	18.33 (20.95)
Re-hospitalization, *n* (%)		20,164 (14.9)	17,638 (14.2)	2,526 (22.1)
Average times of re-hospitalization, times		1.24 (0.76)	1.23 (0.73)	1.38 (0.98)
ICU transferal, *n* (%)		10,844 (8.0)	9,118 (7.3)	1,726 (15.1)
Serum sodium level on admission, mmol/L		139.41 (3.48)	139.93 (2.23)	133.81 (7.43)
Average serum sodium level, mmol/L		139.25 (2.73)	139.55 (2.15)	135.98 (5.13)
Minimum serum sodium level, mmol/L		136.91 (3.71)	137.45 (2.99)	131.14 (5.46)
Maximum serum sodium level, mmol/L		141.58 (3.36)	141.66 (2.78)	140.66 (7.01)
Fluctuation range of serum sodium level, mmol/L		4.66 (4.28)	4.21 (3.68)	9.52 (6.69)
Outcome				
Mortality, *n* (%)		1,912 (1.4)	1,088 (0.9)	824 (7.2)
	In-hospital mortality	1,412 (1.0)	785 (0.6)	627 (5.5)
	AMA discharge	500 (0.4)	303 (0.2)	197 (1.7)

Categorical variables are presented as counts and percentages; continuous variables are presented as mean ± SD. AMA, against medical advice; ICU, intensive care unit.

Among these mortality cases, 824 (7.2%) patients had dysnatremia on admission, and 1,088 (0.9%) patients were normonatremia on admission.

### 3.2 Impact of sodium fluctuation level during hospitalization on outcomes

Sodium fluctuation level during hospitalization showed a dose-dependent association with increased mortality in general hospitalized patients. The mortality rate of group G1 to G6 were 372 (0.4%), 554 (2.4%), 475 (10.2%), 274 (17.7%), 159 (29.3%), and 85 (26.6%), respectively, *p* trend <0.001, as shown in Table 2. As Supplementary Table S2 showed that sodium fluctuation level was moderately correlated with length of hospital stays (*r* = 0.45, *p* < 0.001), and was weakly correlated with age (*r* = 0.09, *p* < 0.001) and CCI (*r* = 0.13, *p* < 0.001). Therefore, we adjusted age, sex, CCI, and length of hospital stays in model 1. The ORs of group G2 to G6 were 5.92 (95% CI 5.16–6.79), 26.45 (95% CI 22.68–30.86), 50.71 (95% CI 41.78–61.55), 104.38 (95% CI 81.57–133.58), and 157.64 (95% CI 112.83–220.24), respectively, *p* trend <0.001. Moreover, both normonatremia patients and dysnatremia patients on admission showed the dose-dependent associations similar to general hospitalized patients. After adjusting age, sex, CCI, and length of hospital stay, the ORs of group G2 to G6 in normonatremia patients on admission were 6.90 (95% CI 5.48–7.68), 34.26 (95% CI 28.42–41.29), 71.85 (95% CI 56.23–91.81), 121.87 (95% CI 85.26–174.18), and 226.55 (95% CI 136.54–375.90), respectively, *p* trend <0.001; the ORs of group G2 to G6 in dysnatremia patients on admission were 2.06 (95% CI 1.63–2.60), 5.34 (95% CI 4.15–6.88), 8.66 (95% CI 6.43–11.66), 20.77 (95% CI 14.70–29.34), and 26.02 (95% CI 16.62–40.73), respectively, *p* trend <0.001.

**Table 2:** The association between sodium fluctuation level during hospitalization and mortality based on generalized estimated equations.

	G1 group	G2 group	G3 group	G4 group	G5 group	G6 group	*P* trends
Sodium fluctuation level during hospitalization, mmol/L	0–6.0	6.0–12.0	12.0–18.0	18.0–24.0	24.0–30.0	> 30.0	
**General hospitalized patients**							
*N*, *n*	105,808	22,702	4,676	1,545	519	232	
Mortality, *n* (%)	372 (0.4)	554 (2.4)	475 (10.2)	274 (17.7)	152 (29.3)	85 (26.6)	<0.001
Model 1^a^	1.00	5.92 (5.16–6.79)	26.45 (22.68–30.86)	50.71 (41.78–61.55)	104.38 (81.57–133.58)	157.64 (112.83–220.24)	<0.001
Model 2^b^	1.00	6.32 (5.50–7.26)	26.76 (22.89–31.29)	45.40 (37.39–55.12)	83.42 (564.51–107.87)	112.55 (79.67–159.01)	<0.001
**Normonatremia patients on admission**							
*N*, *n*	101,471	18,394	3,031	843	228	89	
Mortality, *n* (%)	239 (0.2)	331 (1.8)	279 (9.2)	147 (17.4)	58 (25.4)	34 (38.2)	<0.001
Model 1^a^	1.00	6.90 (5.48–7.68)	34.26 (28.42–41.29)	71.85 (56.23–91.81)	121.87 (85.26–174.18)	226.55 (136.54–375.90)	<0.001
Model 2^b^	1.00	7.09 (5.97–8.40)	35.44 (29.28–42.90)	63.81 (49.78–81.80)	92.05 (63.07–134.33)	147.95 (86.64–252.66)	<0.001
**Dysnatremia patients on admission**							
*N*, *n*	4,337	4,308	1,645	702	291	143	
Mortality, *n* (%)	133 (3.1)	223 (5.2)	196 (11.9)	127 (18.1)	94 (32.3)	51 (35.7)	<0.001
Model 1^a^	1.00	2.06 (1.63–2.60)	5.34 (4.15–6.88)	8.66 (6.43–11.66)	20.77 (14.70–29.34)	26.02 (16.62–40.73)	<0.001
Model 2^b^	1.00	1.89 (1.50–2.40)	4.62 (3.58–5.96)	6.36 (3.66–8.67)	13.56 (9.40–19.54)	14.89 (9.27–23.91)	<0.001

Categorical variables are presented as counts and percentages. Results of models are presented in Odds Ratio and 95% Confidence Interval. Cochran-Armitage test was used to examine trends among categorical variables and odds ratios. ^a^Adjusted with age, sex, length of hospital stays, and Charlson Comorbidities Index. ^b^Adjusted with age, sex, length of hospital stays, myocardial infarction, chronic pulmonary disease, moderate or severe liver disease, moderate or severe renal disease, metastatic solid tumor, serum sodium level on admission and average serum sodium level during hospitalization.

**Figure 2:**
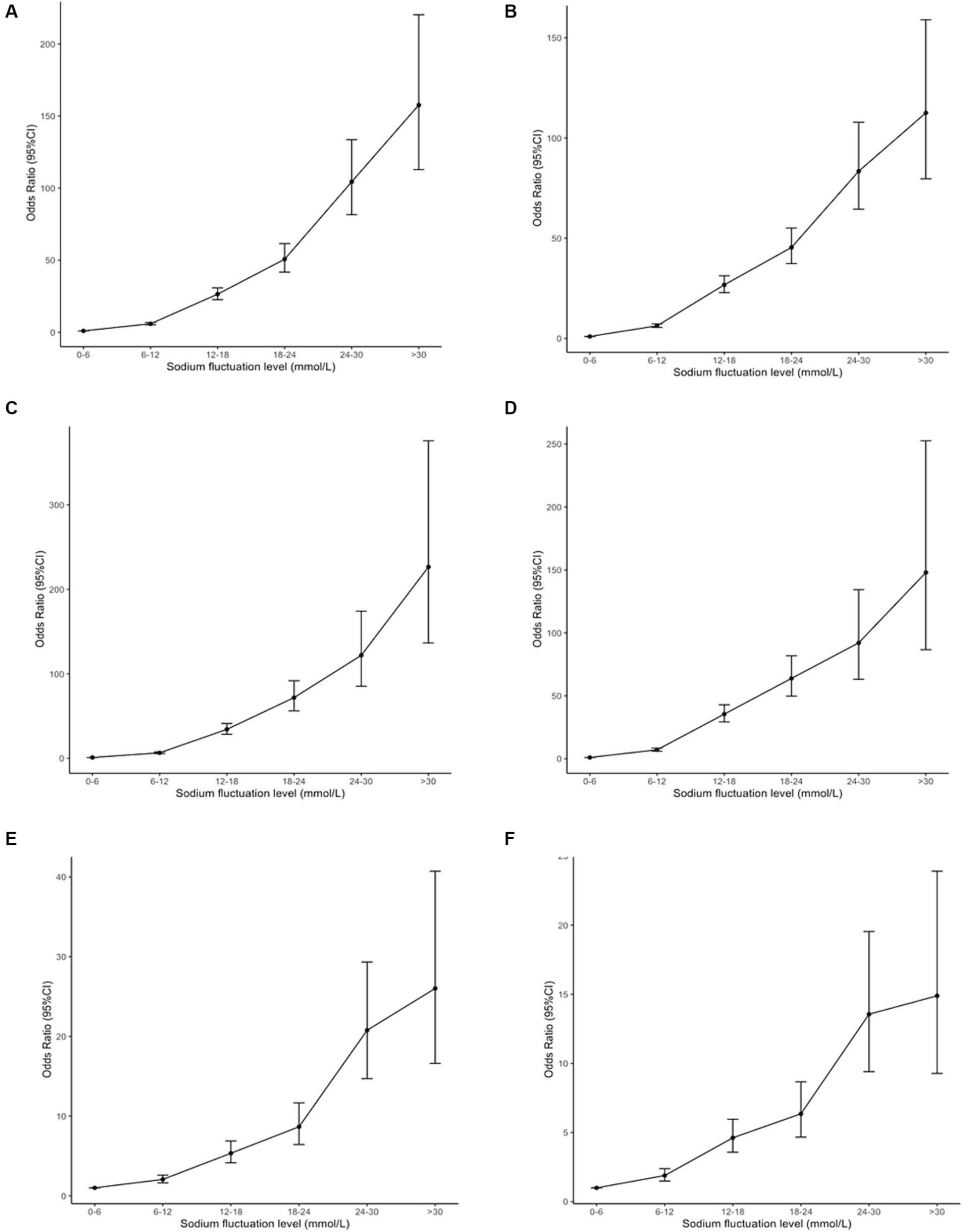
The association between serum sodium fluctuation level during hospitalization (cutoff = 6 mmol/L) and mortality. The Odds Ratios (OR) and 95% Confidence Intervals (CI) were generated based on generalized estimated equations. **(A,C,E)** Showed the OR and 95% CI of sodium fluctuation level during hospitalization (cutoff = 6 mmol/L) after adjustment of age, sex, length of hospital stays, and Charlson Comorbidities Index in general hospitalized patients, normonatremia patients on admission, and dysnatremia patients on admission. **(B,D,F)** Showed the OR and 95% CI of sodium fluctuation level during hospitalization (cutoff = 6 mmol/L) after adjustment of age, sex, length of hospital stays, myocardial infarction, chronic lung disease, moderate-to-severe liver failure, moderate-to-severe kidney failure, metastatic solid tumor, serum sodium level on admission and average serum sodium level during hospitalization in general hospitalized patients, normonatremia patients on admission, and dysnatremia patients on admission. CI, confidence interval.

Figure 2 depicts the dose-dependent association between sodium fluctuation level during hospitalization and mortality in general hospitalized patients, normonatremia patients on admission, and dysnatremia patients on admission. The results of the sensitivity analysis were shown in Supplementary Tables S3, S4, which showed no significant difference with Table 2. We further examined the association between sodium fluctuation level divided by cutoffs of 3 and 10 and mortality. The results were consistent with the results of sodium fluctuation divided by cutoffs of 6, which were summarized in Supplementary Tables S5, S6, Supplementary Figures S1, S2.

### 3.3 The association between sodium fluctuation level during hospitalization, minimum serum sodium level, maximum serum sodium level and mortality

Table 3 demonstrated that sodium fluctuation level during hospitalization was associated with mortality in general hospitalized patients, normonatremia patients on admission, and dysnatremia patients on admission. The ORs of sodium fluctuation level increased per 1 mmol/L were 1.21 (95% CI 1.20–1.22), 1.24 (95% CI 1.23–1.26), and 1.12 (95% CI 1.10–1.14), respectively, after adjusting age, sex, length of hospital stays, and CCI. Additionally, an increase in the maximum serum sodium level was associated with increased mortality in general hospitalized patients (OR 1.21, 95% CI 1.20–1.22), normonatremia patients on admission (OR 1.27, 95% CI 1.25–1.29), and dysnatremia patients on admission (OR 1.11, 95% CI 1.10–1.13), after the adjustment of age, sex, length of hospital stays, and CCI. However, minimum serum sodium level was associated with mortality in only general hospitalized patients and normonatremia patients on admission. After the adjustment of age, sex, length of hospital stays, and CCI, the ORs of minimum serum sodium level decreased per 1 mmol/L were 1.39 (95% CI 1.37–1.41) and 1.46 (95% CI 1.43–1.49), respectively. The sensitivity analysis yielded similar results, as shown in Supplementary Tables S7, S8.

**Table 3:** The association between sodium fluctuation level during hospitalization, minimum serum sodium level, maximum serum sodium level and mortality based on generalized estimated equations.

	Minimum serum sodium level, decrease per 1 mmol/L	*P*-value	Maximum serum sodium level, increase per 1 mmol/L	*P*-value	Sodium fluctuation level during hospitalization, increase per 1 mmol/L	*P*-value
**General hospitalized patients**						
Model 1^a^	1.39 (1.37–1.41)	<0.001	1.36 (1.34–1.39)	<0.001	1.20 (1.19–1.21)	<0.001
Model 2^b^	1.15 (1.14–1.16)	<0.001	1.21 (1.20–1.22)	<0.001	1.21 (1.20–1.22)	<0.001
**Normonatremia patients on admission**						
Model 1^a^	1.46 (1.43–1.49)	<0.001	1.42 (1.39–1.45)	<0.001	1.24 (1.22–1.25)	<0.001
Model 2^b^	1.19 (1.17–1.21)	<0.001	1.27 (1.25–1.29)	<0.001	1.24 (1.23–1.26)	<0.001
**Dysnatremia patients on admission**						
Model 1^a^	1.18 (1.15–1.21)	<0.001	1.18 (1.16–1.21)	<0.001	1.10 (1.09–1.12)	<0.001
Model 2^b^	0.99 (0.97–1.01)	0.203	1.11 (1.10–1.13)	<0.001	1.12 (1.10–1.14)	<0.001

Categorical variables are presented as counts and percentages. Results of models are presented in Odds Ratio and 95% Confidence Interval. ^a^Adjusted with age, sex, length of hospital stays, myocardial infarction, chronic pulmonary disease, moderate or severe liver disease, moderate or severe renal disease, and metastatic solid tumor. ^b^Adjusted with age, sex, length of hospital stays, and Charlson Comorbidities Index.

### 3.4 Sodium fluctuation level during hospitalization predict mortality

**Figure 3:**
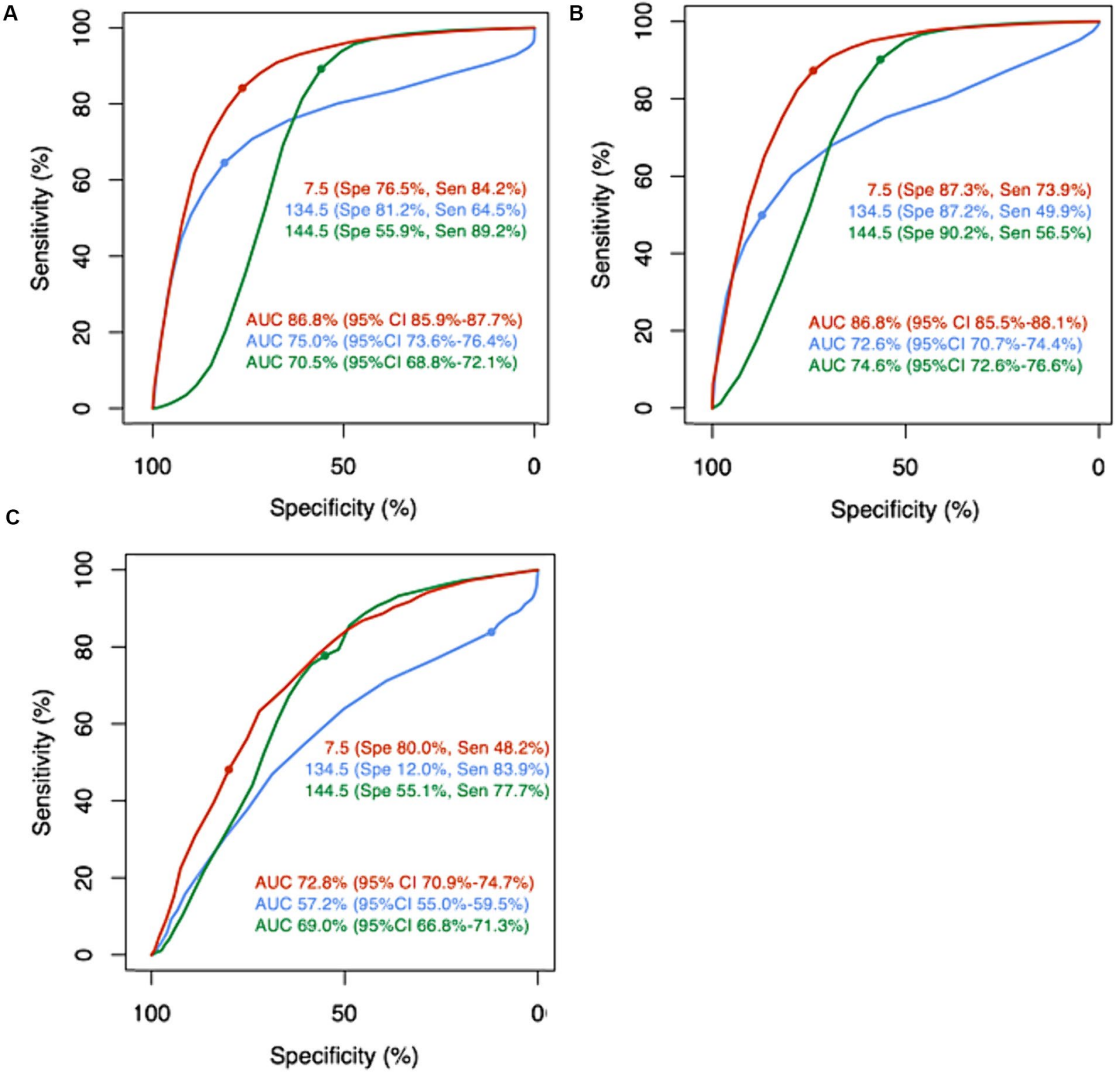
The receiver operating character curve of minimum serum sodium level (blue curve), maximum serum sodium level (green curve), and sodium fluctuation level during hospitalization (red curve) in predicting mortality. The cutoffs of minimum serum sodium level, maximum serum sodium level, and sodium fluctuation level during hospitalization were determined based on the optimal cutoffs in general hospitalized patients. **(A–C)** Showed the Receiver Operating Character Curves in general hospitalized patients, normonatremia patients on admission, and dysnatremia patients on admission, respectively. AUC, area under curve; CI, confidence internal; Sen, sensitivity; Spe, specificity.

ROC curve analysis was performed to calculate the optimal cutoff values and the AUCs of minimum serum sodium level, maximum serum sodium level, and sodium fluctuation level during hospitalization in general hospitalized patients, as shown in Figure 3A. The AUC of sodium fluctuation level during hospitalization was 0.868 (95% CI 0.859–0.877), which was significantly higher than minimum serum sodium level (AUC 0.750, 95% CI 0.736–0.764, *p* < 0.001) and maximum serum sodium level (AUC 0.705, 95% CI 0.688–0.721, *p* < 0.001). In addition, the AUCs of sodium fluctuation level were 0.868 (95% CI 0.855–0.881) in normonatremia patients on admission and 0.728 (95% CI 0.709–0.747) in dysnatremia patients on admission, which were also significantly higher than the AUCs of minimum and maximum serum sodium level in both normonatremia and dysnatremia patients on admission.

The optimal cutoff point of sodium fluctuation level during hospitalization was 7.5 mmol/L in general hospitalized patients, with a sensitivity of 76.5% and a specificity of 84.2%. The cutoff points of minimum and maximum serum sodium levels were 134.5 mmol/L and 144.5 mmol/L, respectively. These cutoff points were further examined in normonatremia patients and dysnatremia patients on admission, as depicted in Figures 3B,C. The cutoff point of sodium fluctuation level (7.5 mmol/L) had a sensitivity of 73.9%, a specificity of 87.3% in normonatremia patients on admission, and a sensitivity of 48.2%, a specificity of 80.0% in dysnatremia patients on admission. Compared to patients with sodium fluctuation level below 7.5 mmol/L, patients with sodium fluctuation level higher than 7.5 mmol/L had a significantly increased mortality rate in general hospitalized patients (0.4% vs. 6.5%, *p* < 0.001), normonatremia patients on admission (0.3% vs. 4.9%, *p* < 0.001), and dysnatremia patients on admission (3.1% vs. 10.7%, *p* < 0.001), as shown in Table 4.

**Table 4:** Outcomes of patients divided by cutoffs of sodium fluctuation level during hospitalization recognized by receiver operator characteristic curves in general hospitalized patients.

	General hospitalized patients			Normonatremia patients on admission			Dysnatremia patients on admission		
	< 7.5 mmol/L	> 7.5 mmol/L	*P*-value	< 7.5 mmol/L	> 7.5 mmol/L	*P*-value	< 7.5 mmol/L	> 7.5 mmol/L	*P*-value
*N*	112,905	22,577		107,634	16,422		5,271	6,155	
Mortality, *n* (%)	449 (0.4)	1,463 (6.5)	<0.001	284 (0.3)	804 (4.9)	<0.001	165 (3.1)	659 (10.7)	<0.001
In-hospital mortality, *n* (%)	265 (0.2)	1,147 (5.1)	<0.001	143 (0.1)	642 (3.9)	<0.001	122 (2.3)	505 (8.2)	<0.001
AMA discharge, *n* (%)	184 (0.2)	316 (1.4)	<0.001	141 (0.1)	162 (1.0)	<0.001	43 (0.8)	154 (2.5)	<0.001

Categorical variables are presented as counts and percentages. Results of models are presented in Odds Ratio and 95% Confidence Interval. AMA, against medical advice.

The sensitivity analysis of ROC curves demonstrated that the AUC of sodium fluctuation was higher than that of minimum and maximum sodium levels in general hospitalized patients, normonatremia patients on admission, and dysnatremia patients on admission. The results of the sensitivity analysis are presented in Supplementary Figures S3, S4, which align with the findings in Figure 3. Furthermore, the sensitivity analysis of the optimal cutoff value showed that, compared to patients with a sodium fluctuation level below 7.5 mmol/L, patients with a sodium fluctuation level higher than 7.5 mmol/L had a significantly increased mortality rate in general hospitalized patients, normonatremia patients on admission, and dysnatremia patients on admission. These results are presented in Supplementary Tables S9, S10, which are consistent with the findings in Table 4. The result of Briers score and its sensitivity analysis were shown in Supplementary Tables S11–S13. The result of calibration metric and its sensitivity analysis were shown in Supplementary Figures S5–S7.

## 4 Discussion

In this study, we examined the dose-dependent association between sodium fluctuation level during hospitalization and mortality in general hospitalized patients, normonatremia patients on admission, and dysnatremia patients on admission. We evaluated the value of sodium fluctuation level during hospitalization in dysnatremia management, which could be used as a marker to develop a single parameter system for predicting adverse outcomes in general hospitalized patients with acceptable accuracy, sensitivity, and specificity.

The study was conducted in a large retrospective cohort. Dysnatremia is the most common electrolyte disorder and is independently associated with adverse outcomes (3, 5). The serum sodium level is the most commonly used parameter in dysnatremia management. Previous studies suggested that compared to patients with simple hyponatremia or simple hypernatremia, patients with mixed dysnatremia had a higher risk of mortality (9). In addition, patients with serum sodium level between reference ranges (135 mmol/L-145 mmol/L) are regarded as low-risk patients. However, recent studies have suggested that sodium fluctuations were associated with mortality even within sodium reference ranges (10). Therefore, using serum sodium level as a management parameter has several limitations in identifying high-risk patients and providing precise management.

Sodium fluctuation level during hospitalization may be a better parameter in dysnatremia management, considering the clinical significance of mixed dysnatremia and sodium fluctuations within the normal range. Sodium fluctuation level during hospitalization was the difference between the maximum and minimum serum sodium levels (12, 13). Previous studies indicated that sodium fluctuation level below 6.0 mmol/L was treated as safe and was associated with a relatively low risk of death (10). Therefore, we divided patients into 6 groups (group G1-G6) to examine the dose-dependent relationship between sodium fluctuation level during hospitalization and mortality. After multivariable analysis, the ORs of group G2 to G6 in general hospitalized patients were 5.92 (95% CI 5.16–6.79), 26.45 (95% CI 22.68–30.86), 50.71 (95% CI 41.78–61.55), 104.38 (95% CI 81.57–133.58), and 157.64 (95% CI 112.83–220.24), respectively, *p* trend <0.001. The results were similar in normonatremia patients on admission. Patients admitted with dysnatremia also demonstrated a similar dose-dependent association. However, the ORs of group G2 to G6 were lower in these patients due to the independent association between dysnatremia on admission and poor prognosis. Our results suggested that sodium fluctuation level during hospitalization had a dose-dependent association with increased mortality in general hospitalized patients. Therefore, sodium fluctuation level should be paid more attention in clinical practice for accurate identification of high-risk patients.

We evaluated the impact of sodium fluctuation level during hospitalization, minimum and maximum serum sodium levels on adverse outcomes, and further compared the accuracy of the three parameters in predicting mortality. In general hospitalized patients, minimum serum sodium level (OR 1.39, 95% CI 1.37–1.41), maximum serum sodium level (OR 1.21, 95% CI 1.20–1.22), and sodium fluctuation level during hospitalization (OR 1.21, 95% CI 1.20–1.22) were associated with mortality. The ROC results indicated that the AUC of sodium fluctuation level during hospitalization in predicting mortality was significantly higher than the AUC of minimum and maximum serum sodium levels in generalized hospitalized patients, normonatremia patients on admission, and dysnatremia patients on admission. Our results suggested that sodium fluctuation level may better reflect the disease progression (19), which provides more accurate risk stratification in general hospitalized patients.

Our study suggested that sodium fluctuation was another kind of dysnatremia. Previous studies demonstrated that sodium fluctuation was independently associated with increased mortality in normonatremia patients on admission (12, 13, 20, 21). However, the prognostic impact of sodium fluctuation has not been evaluated in dysnatremia patients on admission. Our results showed that sodium fluctuation level during hospitalization had a dose-dependent relationship with increased mortality in general hospitalized patients, normonatremia patients on admission, and dysnatremia patients on admission. Therefore, we suggested that the management of sodium fluctuation level during hospitalization was necessary for decreasing adverse outcomes among general hospitalized patients. Moreover, our study illustrated that sodium fluctuation level could provide a more thorough evaluation in dysnatremia patients as sodium fluctuation level represents the degree of neurohumoral activation (19, 22), which indicates the severity of underlying diseases.

Sodium fluctuation level during hospitalization could be used as a marker to develop a single parameter system for predicting adverse outcomes in general hospitalized patients with acceptable accuracy, sensitivity, and specificity. An important concern of precise management is to prevent clinical deterioration and adverse events in hospitalized patients (23). The ROC analysis revealed that the AUC of sodium fluctuation level in predicting mortality was 0.868 in generalized hospitalized patients. The optimal cut-off point was 7.5 mmol/L with a sensitivity of 76.5% and a specificity of 84.2%. The single parameter system using sodium fluctuation level as the marker performed better than previously reported single parameter systems and multiple parameter weighting systems (24, 25). Our results suggested that the clinical significance of sodium fluctuation should be emphasized in clinical practice. Sodium fluctuation level during hospitalization is a reliable marker for predicting mortality in patients with normonatremia on admission, which provides the basis for precise management in dysnatremia.

Our study has several limitations. First, the retrospective cohort study collected data from a single medical center. The medical center may differ from other hospitals concerning disease spectrum and treatment routine. Therefore, the validity and generalizability of our results need further validation in external cohorts. Second, as this study is an observational study, iatrogenic factors related to sodium fluctuations such as fluid management were not included. Third, sodium fluctuation level during hospitalization was defined as the difference between the maximum and minimum serum sodium levels. However, due to the lack of data on treatment, we were unable to identify sodium fluctuations caused by the correction of dysnatremia or the deterioration of diseases. The clinical significance of sodium fluctuation level during hospitalization needs further validation in patients with treatment data.

## 5 Conclusion

In summary, we determined that sodium fluctuation level during hospitalization had a dose-dependent association with an increased mortality rate in general hospitalized patients, normonatremia patients on admission, and dysnatremia patients on admission. Sodium fluctuation level during hospitalization patients could be used as a marker to develop a single parameter system for predicting adverse outcomes in general hospitalized patients with acceptable accuracy, sensitivity, and specificity.

## Data availability statement

The datasets used in this study are available from the corresponding author on reasonable request.

## Ethics statement

The studies involving humans were approved by Ethics Committee of Peking Union Medical College Hospital, Chinese Academy of Medical Sciences (approval number: S-k1272, approval date: 9 October 2020). The studies were conducted in accordance with the local legislation and institutional requirements. Written informed consent for participation was not required from the participants or the participants’ legal guardians/next of kin in accordance with the national legislation and institutional requirements.

## Author contributions

SL: Conceptualization, Data curation, Formal analysis, Methodology, Writing – original draft, Writing – review & editing. LS: Conceptualization, Writing – review & editing, Data curation, Formal analysis, Methodology, Writing – original draft. YZ: Conceptualization, Methodology, Writing – review & editing. QZ: Conceptualization, Data curation, Writing – review & editing. NJ: Conceptualization, Data curation, Writing – review & editing. HZ: Conceptualization, Supervision, Writing – review & editing. SC: Conceptualization, Methodology, Supervision, Writing – original draft, Writing – review & editing. HP: Conceptualization, Supervision, Writing – review & editing.

## Footnotes

^1^ https://www.R-project.org/
