# Supplementary material for: Sodium fluctuation as a parameter in predicting mortality in general hospitalized patients
Source: Front Med (Lausanne). 2024 Jul 16;11:1399638. doi: 10.3389/fmed.2024.1399638 (PMC11286384; doi:10.3389/fmed.2024.1399638)
Supplement: Supplementary file 1 [file Data_Sheet_1.docx]

Supplementary Material

# Appendices

## Measurement of serum sodium level

Serum sodium level was measured by the laboratory department of Peking Union Medical College Hospital, Chinese Academy of Medical Sciences, and used the indirect ion selection electrode method (Routine: Beckman AU5800 automatic Biochemical Analyzer, Beckman Coulter Co., USA; Emergency: Siemens Dimension EXL Automatic Biochemical Analyzer, Siemens Medical Systems, Germany). The laboratory of Peking Union Medical College Hospital, Chinese Academy of Medical Sciences, conducted indoor quality control on Beckman AU5800 automatic biochemical analyzer twice a day, and Siemens Dimension EXL automatic biochemical analyzer 3 times a day to monitor the precision of serum sodium routine work in our laboratory, to evaluate the reliability of serum sodium test results of clinical samples. The results of serum sodium quality control of the laboratory in recent 5 years showed that the cumulative coefficient of variation was less than the set value, which met the quality indicators of the laboratory.

According to ISO15189 quality management standard, the laboratory department of Peking Union Medical College Hospital needs to detect different detection systems for the same measurements every 6 months to examine the evaluation results of comparability. Twenty blood sodium samples were compared every six months between the Beckman AU5800 system and the Siemens DimensionEXL system. The comparison results met 1/2 (2%) of the acceptable total error in blood sodium determination in the Inter-room quality Assessment Standard for Clinical Laboratory Centers of the Ministry of Health. Therefore, the results of laboratory tests of serum sodium in the last 5 years can be analyzed uniformly.

## Sensitivity analysis

The sensitivity analysis of this study was compromised of two parts. First, we examine the impact of sodium fluctuation on poor prognosis in in-hospital death, AMA discharge, and non-AMA discharged patients. Patients with in-hospital death were identified as patients with poor prognoses. Then we excluded AMA discharged patients, and the patients who died in the hospital were identified as the patients with adverse outcomes and examined the impact of sodium fluctuation on poor prognosis in in-hospital death and medical-discharged patients. Patients with in-hospital death were identified as patients with poor prognosis

# Supplementary Figures and Tables

Supplementary Figures

A
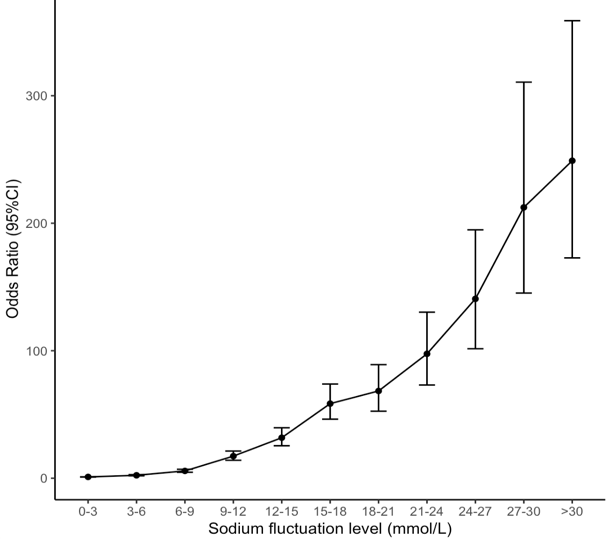
B
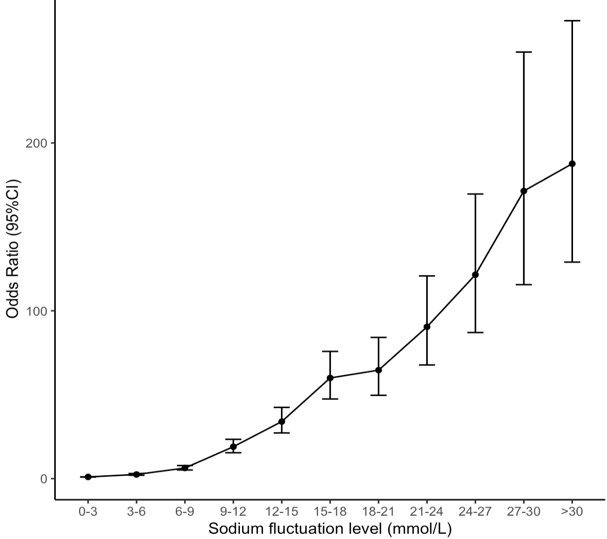


C
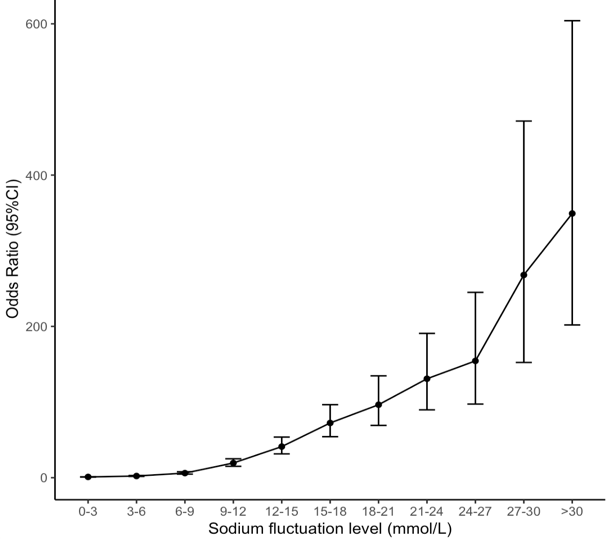
D
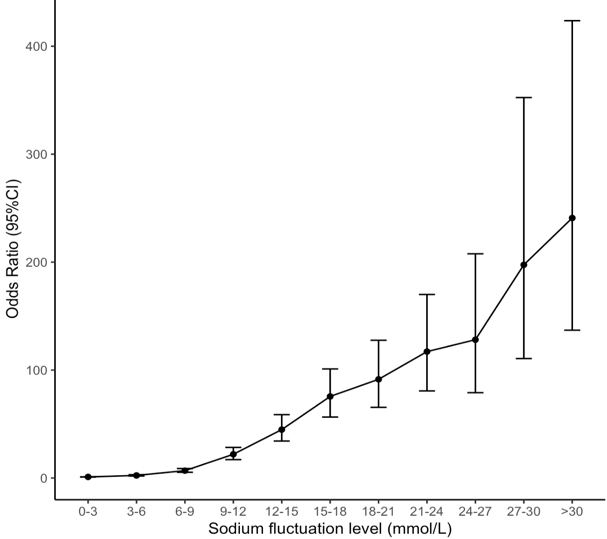


E
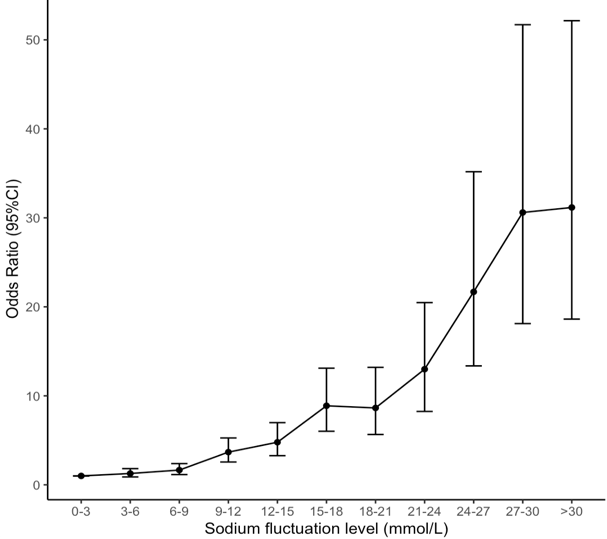
F
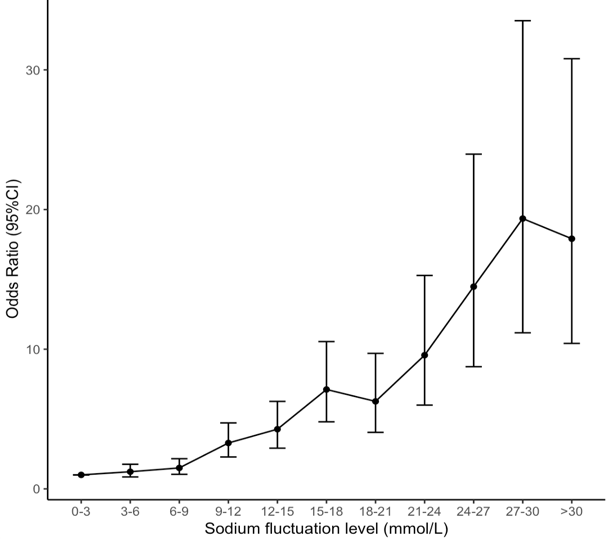


**Figure S1.** The association between serum sodium fluctuation level during hospitalization (cutoff = 3 mmol/L) and mortality.

The Odds Ratios (OR) and 95% Confidence Intervals (CI) were generated based on generalized estimated equations. Figure S1A, C, E showed the OR and 95% CI of fluctuation range of serum sodium level during hospitalization (cutoff = 3 mmol/L) after adjustment of age, sex, length of hospital stays, and Charlson Comorbidities Index in general hospitalized patients, normonatremia patients on admission, and dysnatremia patients on admission. Figure S1B, D, F showed the OR and 95% CI of fluctuation range of serum sodium level during hospitalization (cutoff = 3 mmol/L) after adjustment of age, sex, length of hospital stays, myocardial infarction, chronic lung disease, moderate-to-severe liver failure, moderate-to-severe kidney failure, metastatic solid tumor, serum sodium level on admission and average serum sodium level during hospitalization in general hospitalized patients, normonatremia patients on admission, and dysnatremia patients on admission. Abbreviations: CI: confidence interval.

A
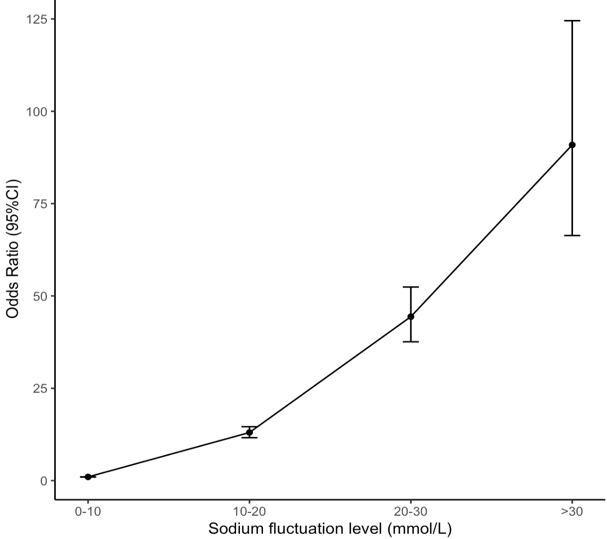
B
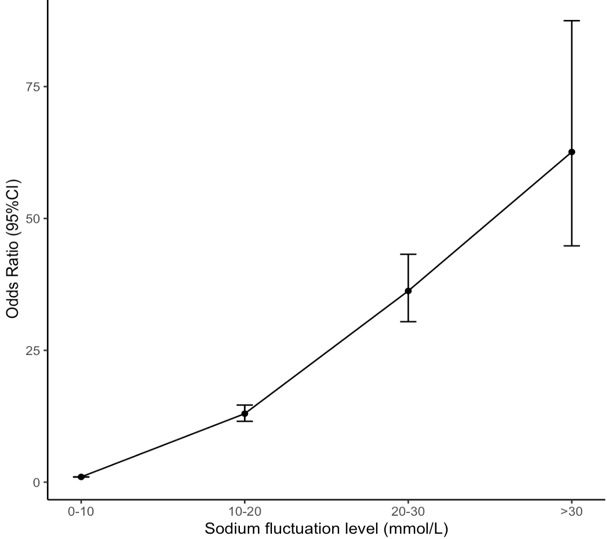


C
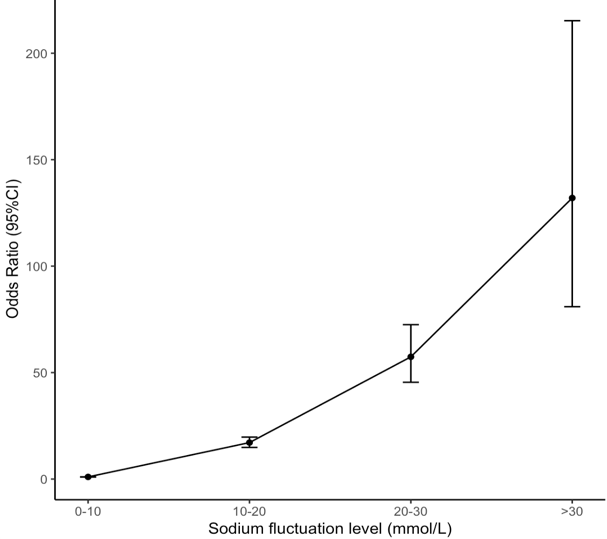
D
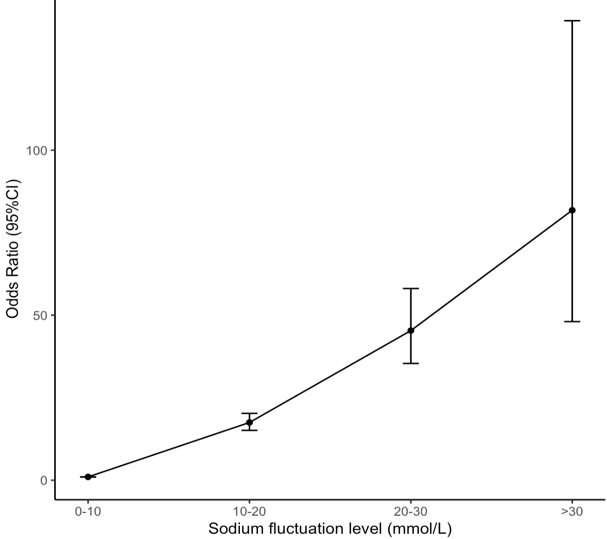


E
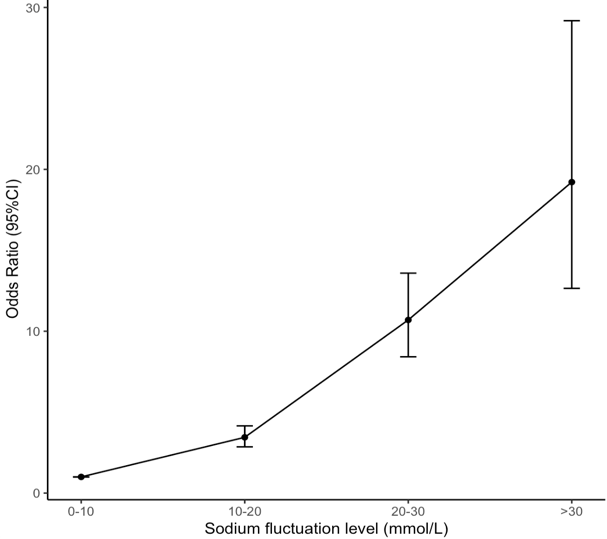
F
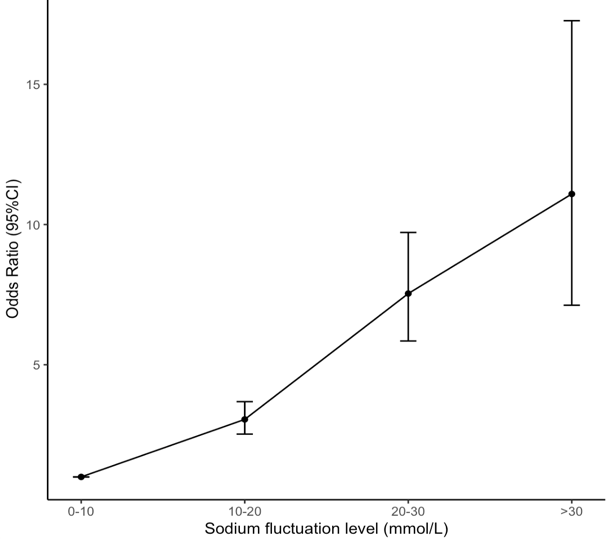


**Figure S2.** The association between sodium fluctuation level during hospitalization (cutoff = 10 mmol/L) and mortality.

The Odds Ratios (OR) and 95% Confidence Intervals (CI) were generated based on generalized estimated equations. Figure S2A, C, E showed the OR and 95% CI of fluctuation range of serum sodium level during hospitalization (cutoff = 10 mmol/L) after adjustment of age, sex, length of hospital stays, and Charlson Comorbidities Index in general hospitalized patients, normonatremia patients on admission, and dysnatremia patients on admission. Figure S2B, D, F showed the OR and 95% CI of fluctuation range of serum sodium level during hospitalization (cutoff = 10 mmol/L) after adjustment of age, sex, length of hospital stays, myocardial infarction, chronic lung disease, moderate-to-severe liver failure, moderate-to-severe kidney failure, metastatic solid tumor, serum sodium level on admission and average serum sodium level during hospitalization in general hospitalized patients, normonatremia patients on admission, and dysnatremia patients on admission. Abbreviations: CI: confidence interval.

A
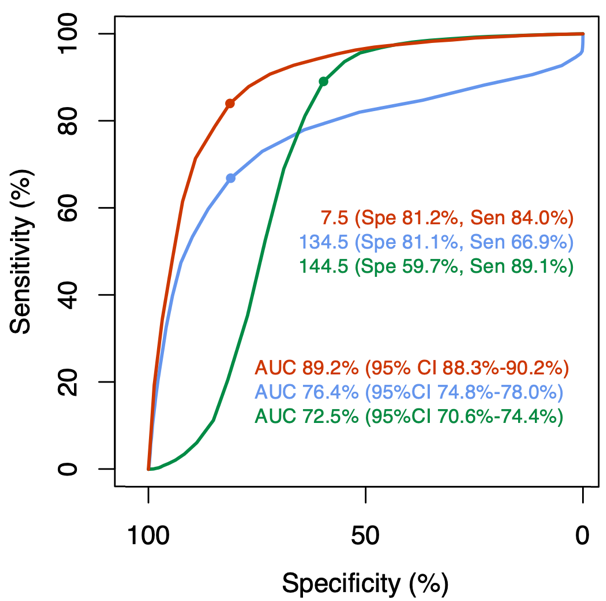
B
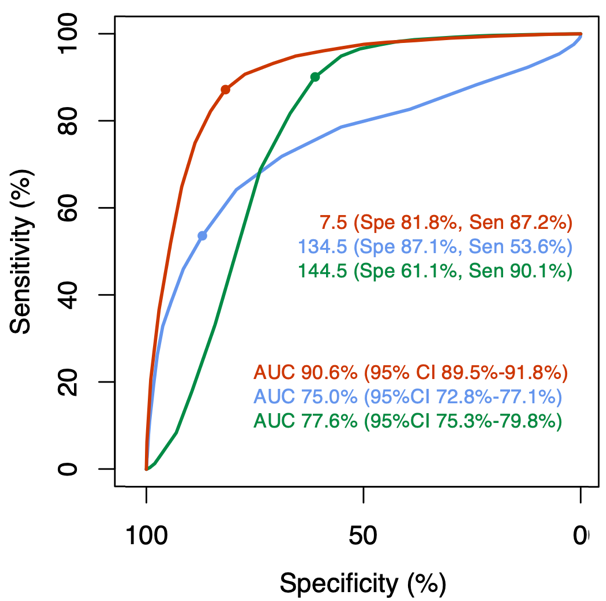


C
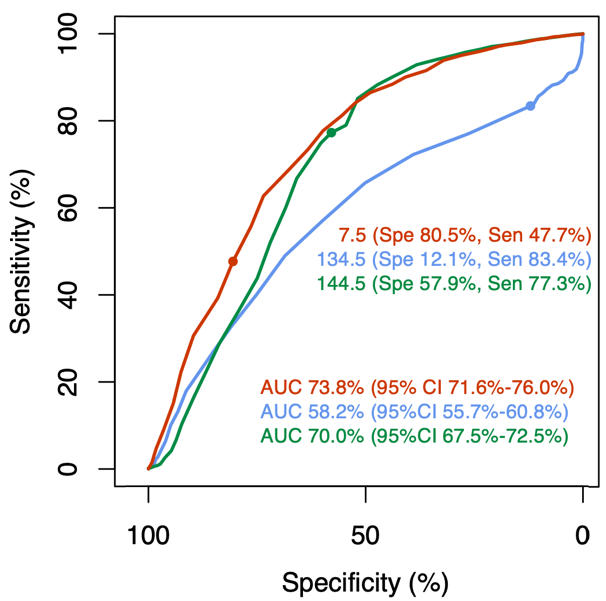


**Figure S3.** The Receiver Operating Character Curve of minimum serum sodium level (blue curve), maximum serum sodium level (green curve) and sodium fluctuation level during hospitalization (red curve) in predicting in-hospital mortality.

The cutoffs of minimum serum sodium level, maximum serum sodium level, and sodium fluctuation level during hospitalization were determined based on the optimal cutoffs of these variables in generalized hospitalized patients. Figure S3A, B, C showed the Receiver Operating Character Curves in general hospitalized patients, normonatremia patients on admission, and dysnatremia patients on admission, respectively.

Abbreviations: AUC: area under curve; CI: confidence internal; Sen: sensitivity; Spe: specificity.

A
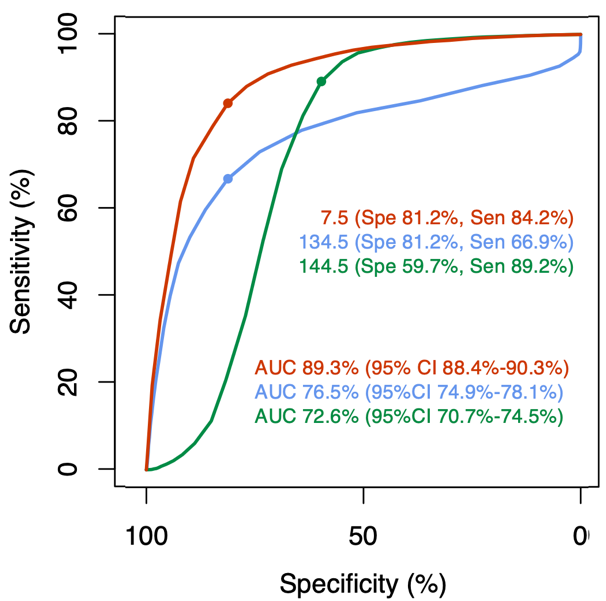
B
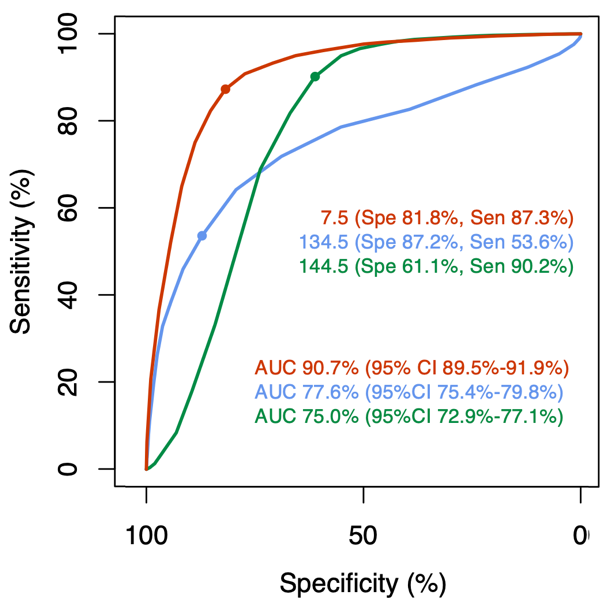


C
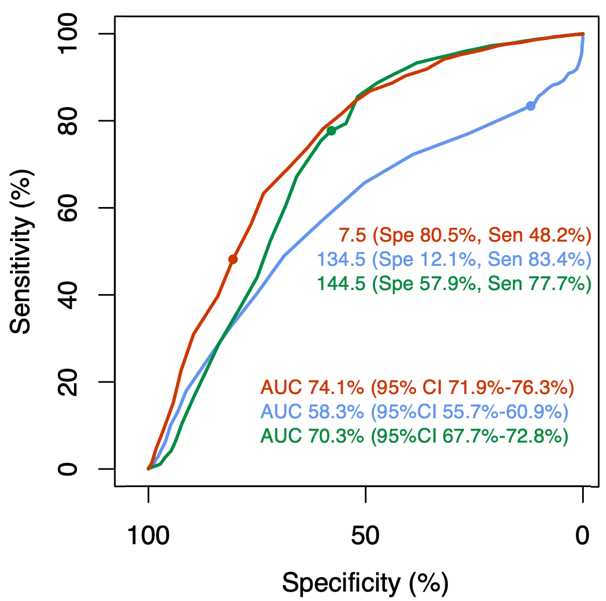


**Figure S4.** The Receiver Operating Character Curve of minimum serum sodium level (blue curve), maximum serum sodium level (green curve) and sodium fluctuation level during hospitalization (red curve) in predicting mortality in non-AMA discharged patients.

The cutoffs of minimum serum sodium level, maximum serum sodium level, and sodium fluctuation level during hospitalization were determined based on the optimal cutoffs of these variables in generalized hospitalized patients. Figure 3A, B, C showed the Receiver Operating Character Curves in general hospitalized patients, normonatremia patients on admission, and dysnatremia patients on admission, respectively.

Abbreviations: AMA: against medical advice; AUC: area under curve; CI: confidence internal; Sen: sensitivity; Spe: specificity

A
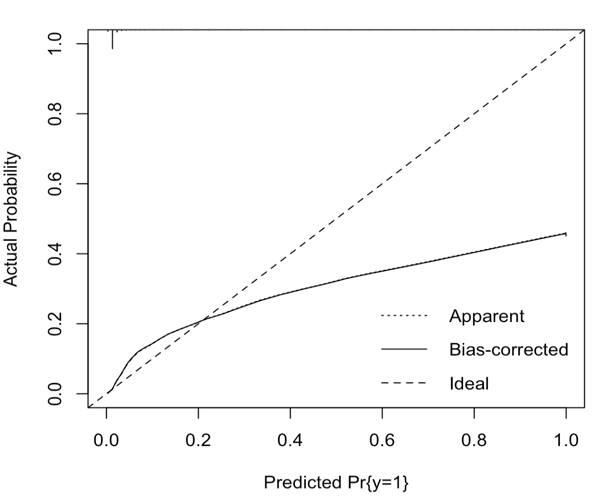
B
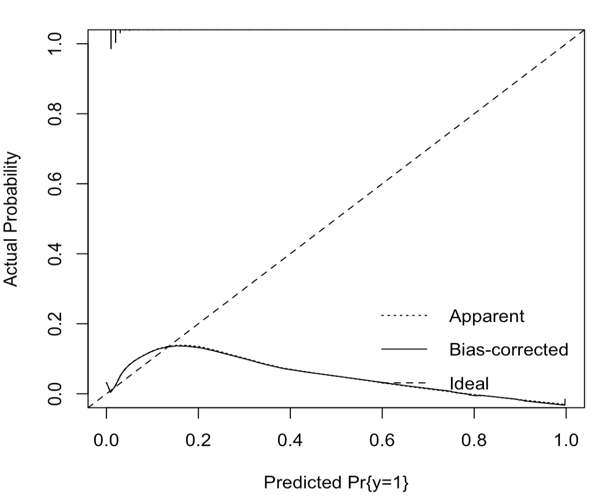
C
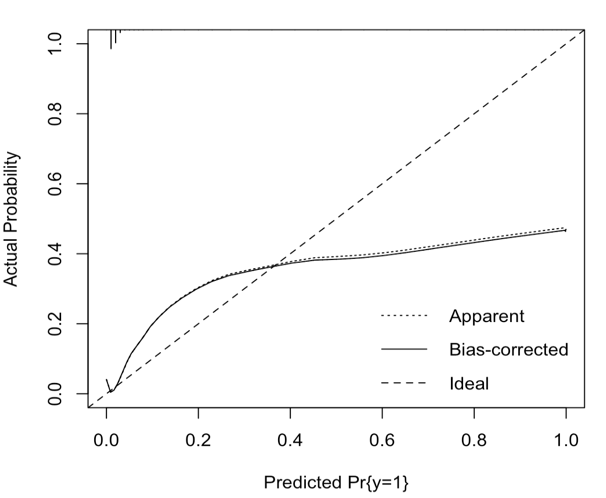


D
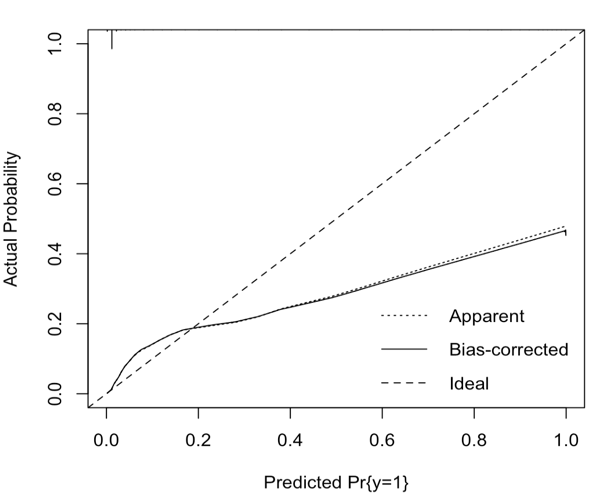
E
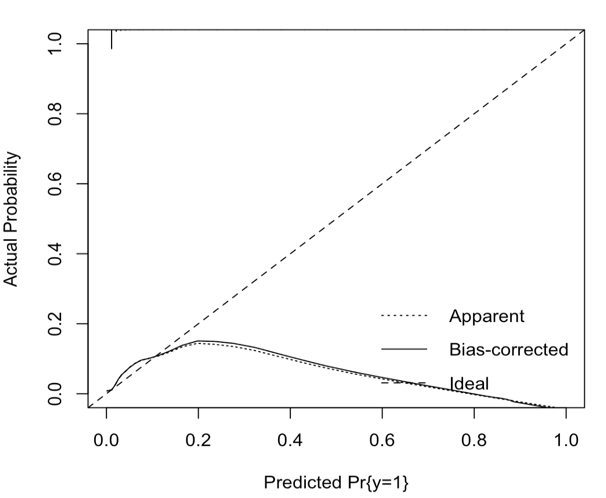
F
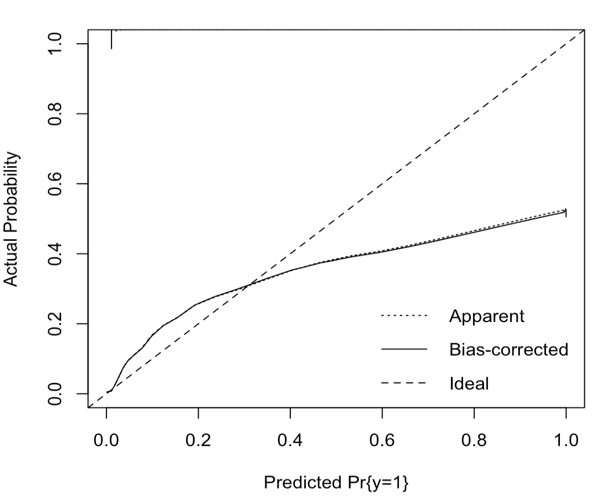


G
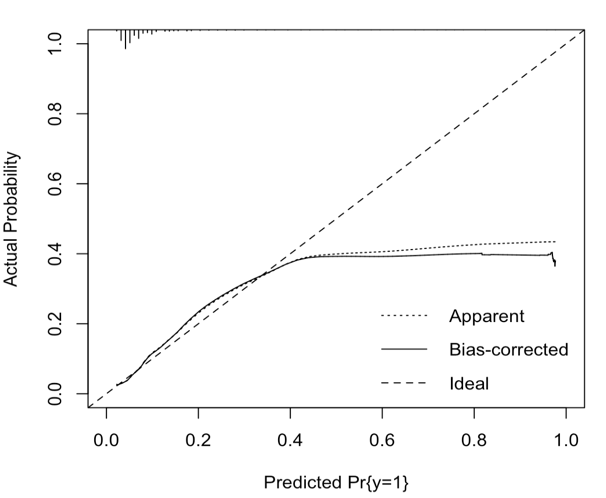
H
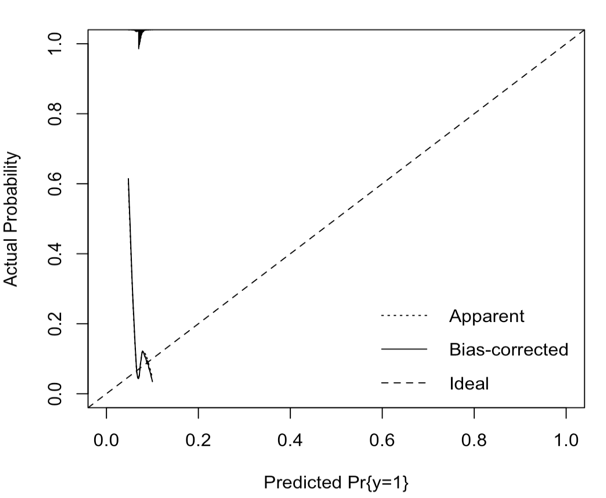
I
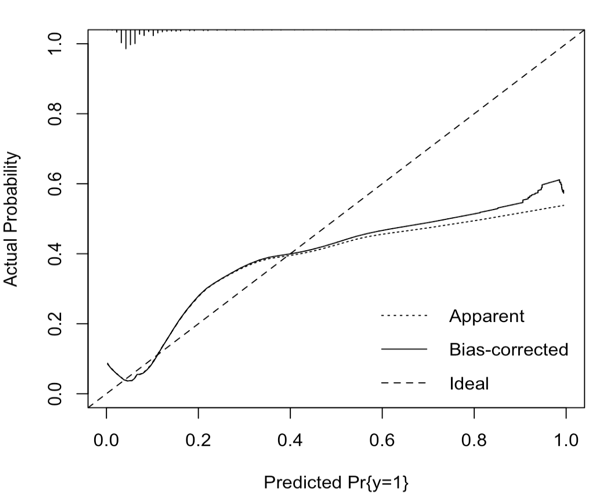


**Figure S5.** The calibration metrics of sodium fluctuation level during hospitalization, minimum serum sodium level, and maximum serum sodium level in predicting mortality.

Figure S5A, B, C showed the calibration metrics of sodium fluctuation level during hospitalization, minimum serum sodium level, and maximum serum sodium level in general hospitalized patients, respectively. Figure S5D, E, F showed the calibration metrics of sodium fluctuation level during hospitalization, minimum serum sodium level, and maximum serum sodium level in normonatremia patients on admission, respectively. Figure S5G, H, I showed the calibration metrics of sodium fluctuation level during hospitalization, minimum serum sodium level, and maximum serum sodium level dysnatremia patients on admission, respectively.

A
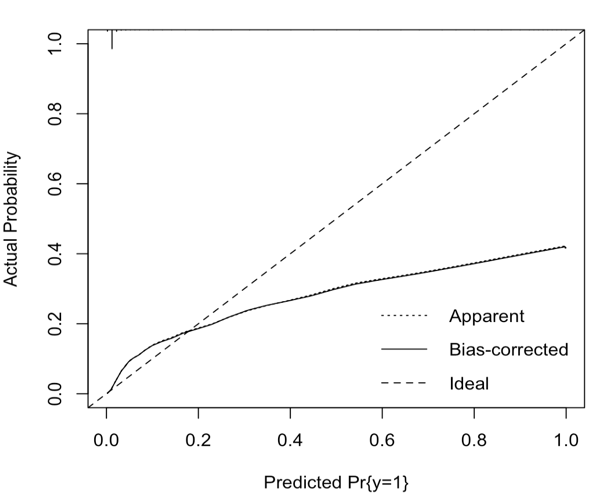
B
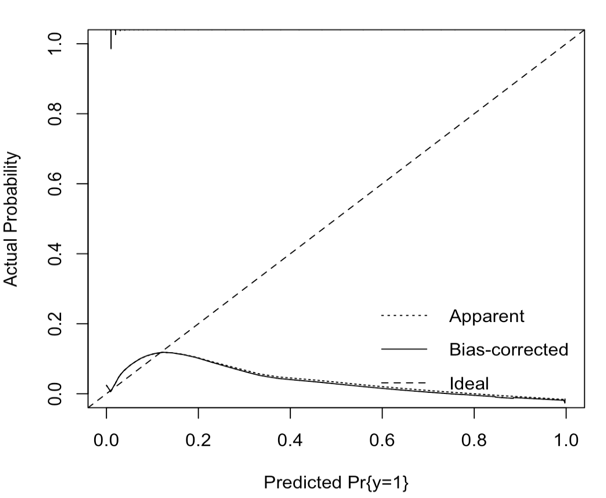
C
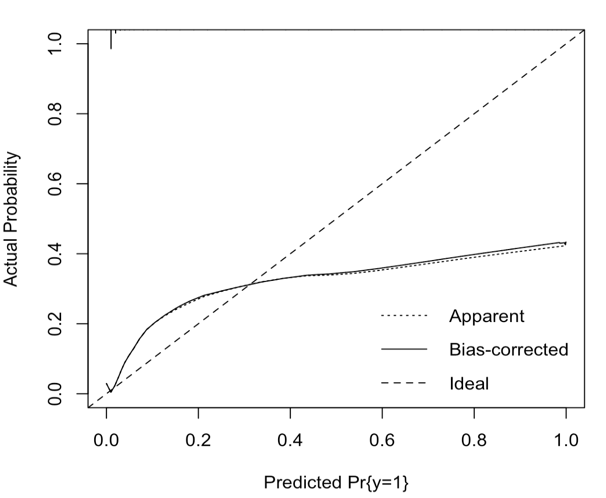

D
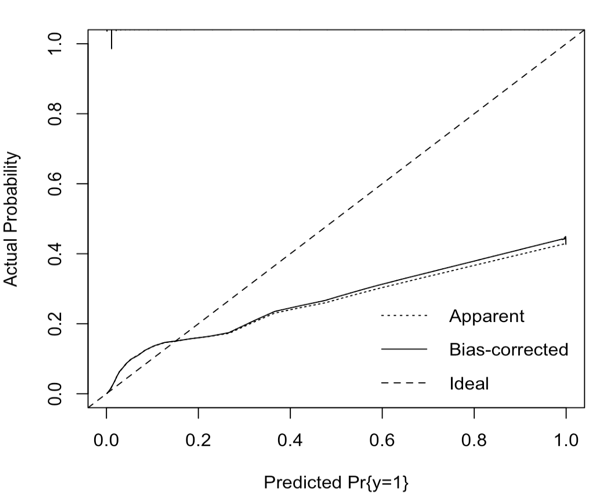
E
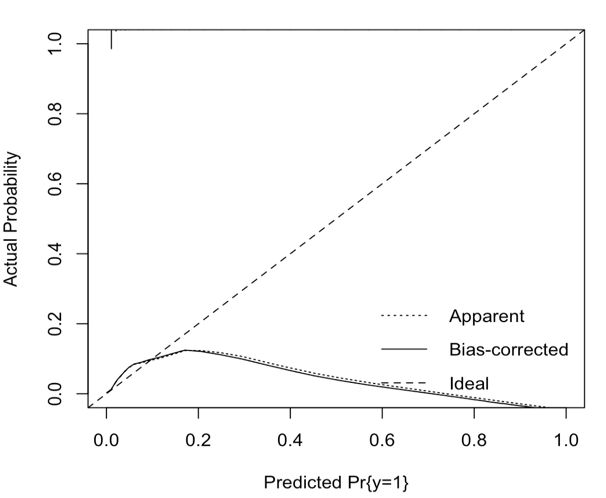
F
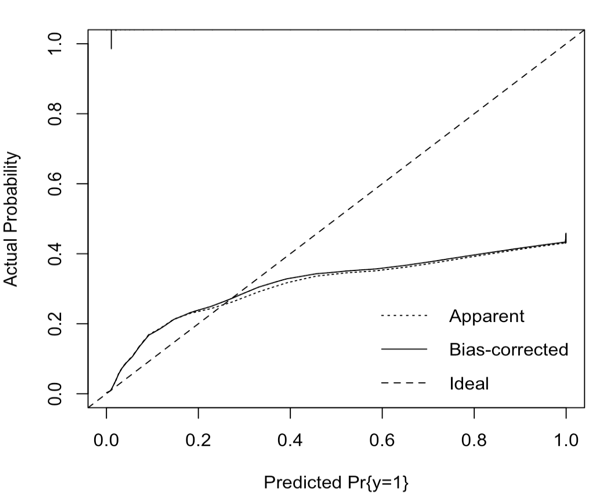


G
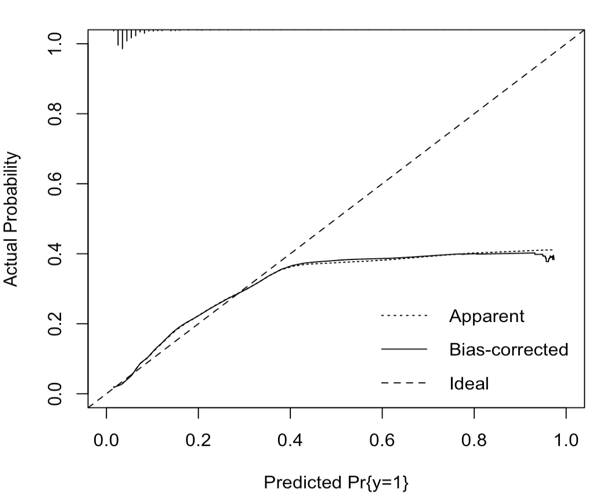
H
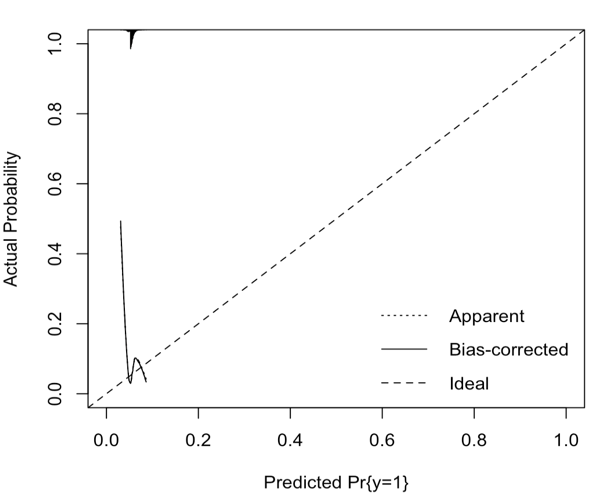
I
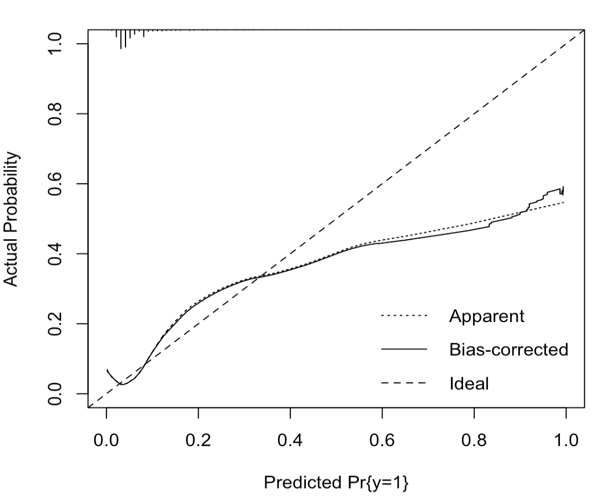


**Figure S6.** The calibration metrics of sodium fluctuation level during hospitalization, minimum serum sodium level, and maximum serum sodium level in predicting in-hospital mortality.

Figure S6A, B, C showed the calibration metrics of sodium fluctuation level during hospitalization, minimum serum sodium level, and maximum serum sodium level in general hospitalized patients, respectively. Figure S6D, E, F showed the calibration metrics of sodium fluctuation level during hospitalization, minimum serum sodium level, and maximum serum sodium level in normonatremia patients on admission, respectively. Figure S6G, H, I showed the calibration metrics of sodium fluctuation level during hospitalization, minimum serum sodium level, and maximum serum sodium level dysnatremia patients on admission, respectively.

A
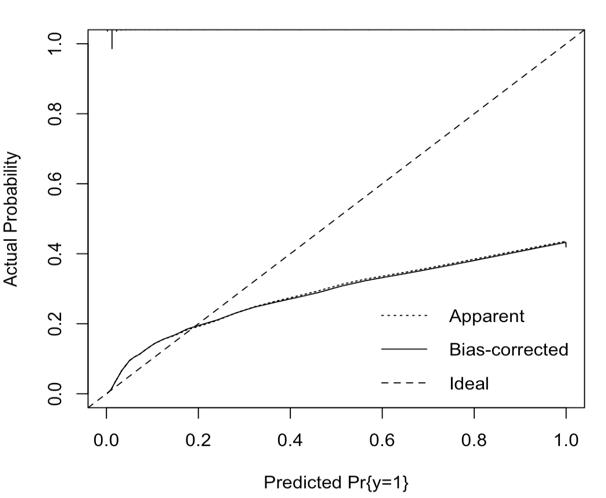
B
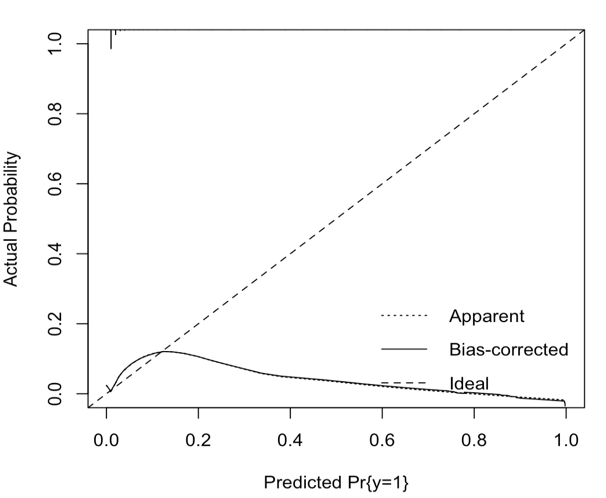
C
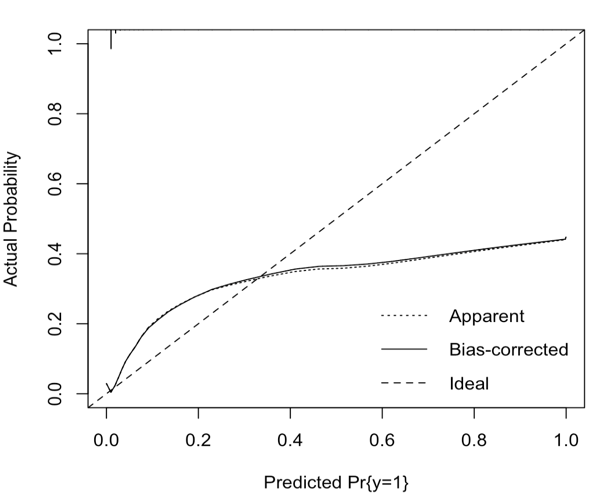


D
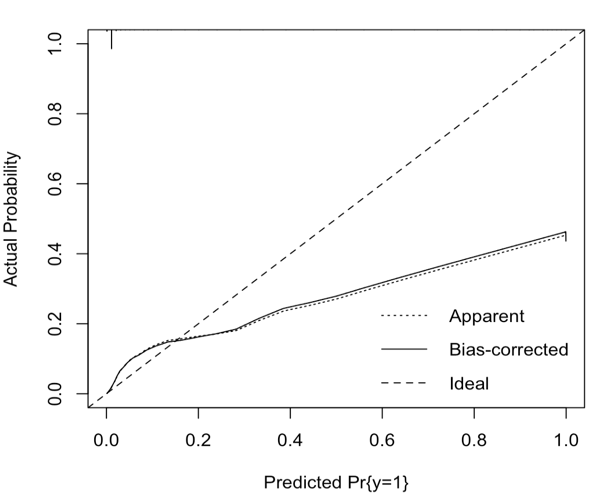
E
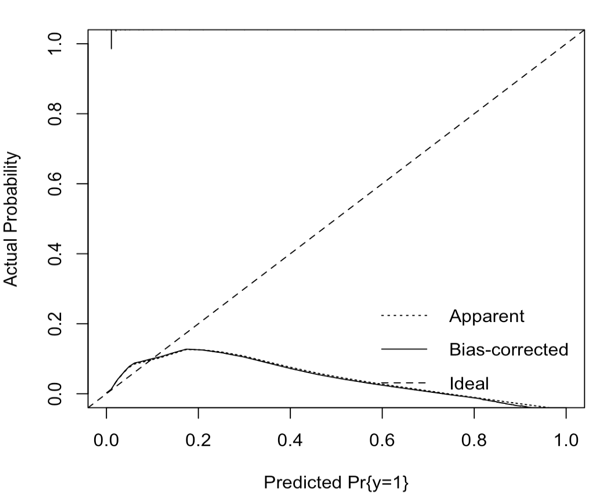
F
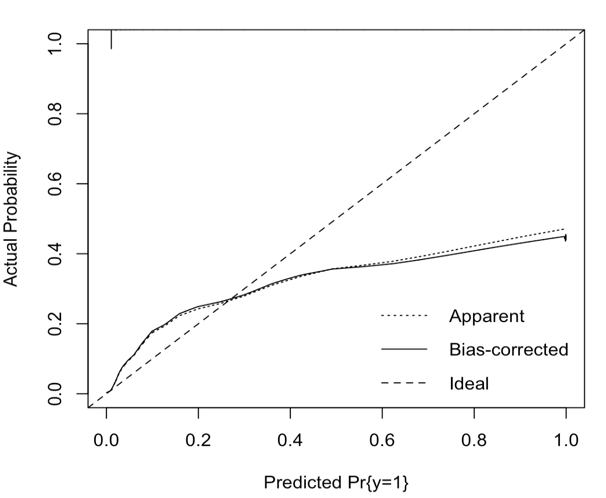


G
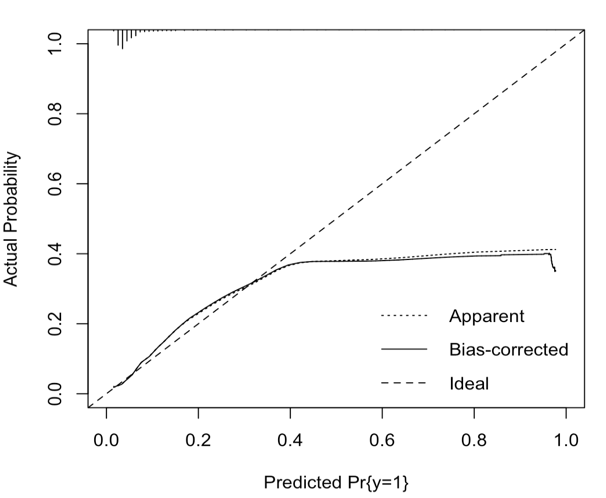
H
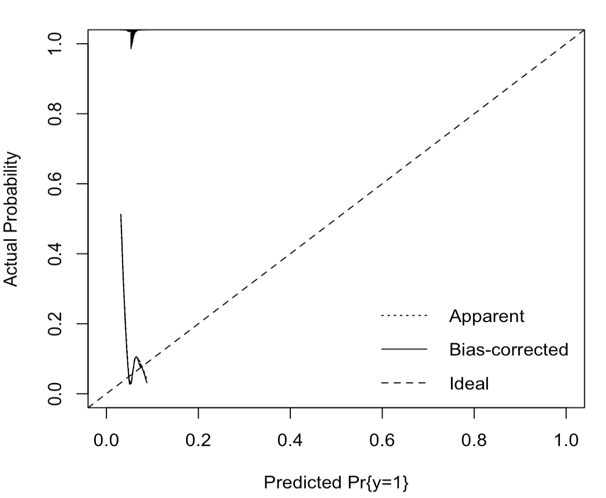
I
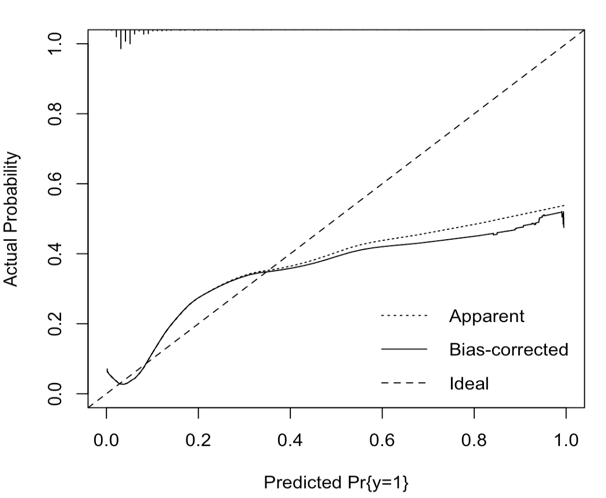


**Figure S7.** The calibration metrics of sodium fluctuation level during hospitalization, minimum serum sodium level, and maximum serum sodium level in predicting mortality in non-AMA discharged patients.

Figure S7A, B, C showed the calibration metrics of sodium fluctuation level during hospitalization, minimum serum sodium level, and maximum serum sodium level in general hospitalized patients, respectively. Figure S7D, E, F showed the calibration metrics of sodium fluctuation level during hospitalization, minimum serum sodium level, and maximum serum sodium level in normonatremia patients on admission, respectively. Figure S7G, H, I showed the calibration metrics of sodium fluctuation level during hospitalization, minimum serum sodium level, and maximum serum sodium level dysnatremia patients on admission, respectively.

Abbreviations: AMA: against medical advice

## For more information on Supplementary Material and for details on the different file types accepted, please see [here](https://www.frontiersin.org/guidelines/author-guidelines#supplementary-material).

Supplementary Tables

**Table S1.** Diagnostic codes in assessing Charlson comorbidity index

| **Comorbidity** | **Points awarded** | **ICD-10 Diagnostic codes** |
| --- | --- | --- |
| Myocardial infarction | 1 | I21, I22, I23 |
| Congestive heart failure | 1 | I50, I11.0, I13.0, I13.2 |
| Peripheral vascular disease | 1 | I70, I71, I72, I73, I74, I77 |
| Cerebrovascular disease | 1 | I60, I61, I62, I63, I64, I65, I66, I67, I68, I69, G45, G46 |
| Dementia | 1 | F00, F01, F02, F03, F05.1, G30 |
| Chronic pulmonary disease | 1 | J40, J41, J42, J43, J44, J45, J46, J47, J60, J61, J62, J63, J64, J65, J66, J67, J68.4, J70.1, J70.3, J84.1, J92.0, J96.1, J98.2, J98.3 |
| Connective tissue disease | 1 | M05, M06, M08, M09, M30, M31, M32, M33, M34, M35, M36, D86 |
| Ulcer disease | 1 | K22,1, K25, K26, K27, K28 |
| Mild liver disease | 1 | B18, K70.0, K70.1, K70.2, K70.3, K70.9, K71, K73, K74, K76.0 |
| Diabetes without end organ damage | 1 | E10.0, E10.1, E10.9, E11.0, E11.1, E11.9 |
| Hemiplegia | 2 | G81, G82 |
| Moderate or severe renal disease | 2 | I12, I13, N00, N01, N02, N03, N04, N05, N11, N14, N17, N18, N19, Q61 |
| Diabetes with end organ damage | 2 | E10.2, E10.3, E10.4, E10.5, E10.6, E10.7, E10.8, E11.2, E11.3, E11.4, E11.5, E11.6, E11.7, E11.8 |
| Tumor without metastasis | 2 | C0, C1, C2, C3, C4, C5, C6, C70, C71, C72, C73, C74, C75 |
| Leukemia | 2 | C91, C92, C93, C94, C95 |
| Lymphoma | 2 | C81, C82, C83, C84, C85, C88, C90, C96 |
| Moderate or severe liver disease | 3 | B15.0, B16.0, B16.2, B19.0, K70.4, K72, K76.6, I85 |
| Metastatic solid tumor | 6 | C76, C77, C78, C79, C80 |
| Acquired immune deficiency syndrome | 6 | B21, B22, B23, B24 |

Abbreviation: ICD-10 International Classification of Diseases, 10th Revision.

**Table S2.** Correlations between hospitalization duration, age, Charlson Comorbidity Index and sodium fluctuation level.

|  | **Sodium fluctuation level, mmol/L** | |
| --- | --- | --- |
|  | **r** | ***P*** |
| Length of hospital stays, day | 0.45 | <0.001 |
| Age, year | 0.09 | <0.001 |
| Charlson Comorbidities Index | 0.13 | <0.001 |

**Table S3.** The association between sodium fluctuation level during hospitalization and in-hospital mortality based on generalized estimated equations.

|  | **G1 group** | **G2 group** | **G3 group** | **G4 group** | **G5 group** | **G6 group** | ***P trends*** |
| --- | --- | --- | --- | --- | --- | --- | --- |
| Sodium fluctuation level during hospitalization, mmol/L | 0-6.0 | 6.0-12.0 | 12.0-18.0 | 18.0-24.0 | 24.0-30.0 | > 30.0 |  |
| General hospitalized patients | | | | | | | |
| N，n | 105808 | 22702 | 4676 | 1545 | 519 | 232 |  |
| Mortality，n (%) | 372 (0.4) | 554 (2.4) | 475 (10.2) | 274 (17.7) | 152 (29.3) | 85 (26.6) | <0.001 |
| Model 1^a^ | 1.00 | 6.87 (5.80-8.15) | 32.48 (27.15-38.86) | 61.69 (49.72-76.54) | 124.88 (95.20-163.81) | 215.95 (152.36-306.06) | <0.001 |
| Model 2^b^ | 1.00 | 7.46 (6.28-8.87) | 33.37 (27.74-40.15) | 54.48 (43.71-67.91) | 97.03 (73.02-128.92) | 149.52 (103.22-216.58) | <0.001 |
| Normonatremia patients on admission | | | | | | | |
| N，n | 101471 | 18394 | 3031 | 843 | 228 | 89 |  |
| Mortality，n (%) | 239 (0.2) | 331 (1.8) | 279 (9.2) | 147 (17.4) | 58 (25.4) | 34 (38.2) | <0.001 |
| Model 1^a^ | 1.00 | 9.26 (7.40-11.58) | 51.52 (40.74-65.16) | 103.31 (77.23-138.20) | 192.53 (128.22-289.10) | 330.69 (192.12-569.20) | <0.001 |
| Model 2^b^ | 1.00 | 10.29 (8.20-12.91) | 54.71 (42.92-69.72) | 92.94 (68.99-125.21) | 147.33 (96.26-225.49) | 218.95 (121.85-393.44) | <0.001 |
| Dysnatremia patients on admission | | | | | | |  |
| N，n | 4337 | 4308 | 1645 | 702 | 291 | 143 |  |
| Mortality，n (%) | 133 (3.1) | 223 (5.2) | 196 (11.9) | 127 (18.1) | 94 (32.3) | 51 (35.7) | <0.001 |
| Model 1^a^ | 1.00 | 1.85 (1.41-2.41) | 5.15 (3.89-6.84) | 8.69 (6.29-12.02) | 18.80 (12.95-27.31) | 31.27 (19.56-49.98) | <0.001 |
| Model 2^b^ | 1.00 | 1.67 (1.27-2.18) | 4.33 (3.26-5.75) | 6.01 (4.28-8.43) | 11.22 (7.54-16.70) | 16.40 9.93-27.09) | <0.001 |

Categorical variables are presented as counts and percentages. Results of models are presented in Odds Ratio and 95% Confidence Interval. Cochran-Armitage test was used to examine trends among categorical variables and odds ratios.

a: Adjusted with age, sex, length of hospital stays, and Charlson Comorbidities Index.

b: Adjusted with age, sex, length of hospital stays, myocardial infarction, chronic pulmonary disease, moderate or severe liver disease, moderate or severe renal disease, metastatic solid tumor, serum sodium level on admission and average serum sodium level during hospitalization.

**Table S4.** The association between sodium fluctuation level during hospitalization and in-hospital mortality in non-AMA discharged patients based on generalized estimated equations.

|  | **G1 group** | **G2 group** | **G3 group** | **G4 group** | **G5 group** | **G6 group** | ***P trends*** |
| --- | --- | --- | --- | --- | --- | --- | --- |
| Sodium fluctuation level during hospitalization, mmol/L | 0-6.0 | 6.0-12.0 | 12.0-18.0 | 18.0-24.0 | 24.0-30.0 | > 30.0 |  |
| General hospitalized patients | | | | | | | |
| N，n | 105808 | 22702 | 4676 | 1545 | 519 | 232 |  |
| Mortality，n (%) | 372 (0.4) | 554 (2.4) | 475 (10.2) | 274 (17.7) | 152 (29.3) | 85 (26.6) | <0.001 |
| Model 1^a^ | 1.00 | 6.93 (5.85-8.22) | 33.37 (27.87-39.95) | 64.99 (52.31-80.75) | 136.05 (103.42-178.97) | 230.05 (161.56-328.29) | <0.001 |
| Model 2^b^ | 1.00 | 7.57 (6.3709.00) | 34.52 (28.67-41.56) | 57.91 (46.40-72.29) | 106.01 (79.52-141.31) | 160.48 (110.33-233.43) | <0.001 |
| Normonatremia patients on admission | | | | | | | |
| N，n | 101471 | 18394 | 3031 | 843 | 228 | 89 |  |
| Mortality，n (%) | 239 (0.2) | 331 (1.8) | 279 (9.2) | 147 (17.4) | 58 (25.4) | 34 (38.2) | <0.001 |
| Model 1^a^ | 1.00 | 9.32 (7.45-11.65) | 52.61 (41.58-66.55) | 108.60 (81.09-145.44) | 202.83 (134.74-305.35) | 360.62 (206.90-628.56) | <0.001 |
| Model 2^b^ | 1.00 | 10.40 (8,29-13.05) | 56.27 (44.12-71.75) | 99.35 (73.80-133.93) | 155.40 (101.16-238.72) | 242.31 (133.13-441.05) | <0.001 |
| Dysnatremia patients on admission | | | | | | |  |
| N，n | 4337 | 4308 | 1645 | 702 | 291 | 143 |  |
| Mortality，n (%) | 133 (3.1) | 223 (5.2) | 196 (11.9) | 127 (18.1) | 94 (32.3) | 51 (35.7) | <0.001 |
| Model 1^a^ | 1.00 | 1.87 (1.43-2.45) | 5.34 (4.02-7.09) | 9.12 (6.58-12.65) | 20.92 (14.33-30.55) | 32.74 (20.34-52.68) | <0.001 |
| Model 2^b^ | 1.00 | 1.70 (1.30-2.22) | 4.47 (3.35-5.94) | 6.27 (4.46-8.82) | 12.44 (8.31-18.62) | 17.11 (10.31-28.39) | <0.001 |

Categorical variables are presented as counts and percentages. Results of models are presented in Odds Ratio and 95% Confidence Interval. Cochran-Armitage test was used to examine trends among categorical variables and odds ratios.

a: Adjusted with age, sex, length of hospital stays, and Charlson Comorbidities Index.

b: Adjusted with age, sex, length of hospital stays, myocardial infarction, chronic pulmonary disease, moderate or severe liver disease, moderate or severe renal disease, metastatic solid tumor, serum sodium level on admission and average serum sodium level during hospitalization.

**Table S5.** The association between sodium fluctuation level (cutoff = 3 mmol/L) and mortality based on generalized estimated equations.

|  | **Sodium fluctuation level** | | | | | | | | | | | ***P trend*** |
| --- | --- | --- | --- | --- | --- | --- | --- | --- | --- | --- | --- | --- |
|  | 0-3.0 mmol/L | 3.0-6.0 mmol/L | 6.0-9.0 mmol/L | 9.0-12.0 mmol/L | 12.0-15.0 mmol/L | 15.0-18.0 mmol/L | 18.0-21.0 mmol/L | 21.0-24.0 mmol/L | 24.0-27.0 mmol/L | 27.0-30.0 mmol/L | > 30.0 mmol/L |  |
| General hospitalized patients | | | | | | | | | | | |  |
| N，n | 65929 | 39879 | 16255 | 6447 | 3021 | 1655 | 973 | 572 | 339 | 180 | 232 |  |
| Mortality，n (%) | 149 (0.2) | 223 (0.6) | 250 (1.5) | 304 (4.7) | 246 (8.1) | 229 (13.8) | 153 (15.7) | 121 (21.2) | 90 (26.5) | 62 (34.4) | 85 (36.6) | <0.001 |
| Model 1^a^ | 1.00 | 2.27 (1.85-2.79) | 5.71 (4.64-7.02) | 17.31 (14.06-21.30) | 31.74 (25.46-39.58) | 58.48 (46.31-73.86) | 68.42 (52.56-89.06) | 97.56 (73.08-130.23) | 140.67 (101.56-194.84) | 212.41 (145.23-194.84) | 249.02 (172.79-358.88) | <0.001 |
| Model 2^b^ | 1.00 | 2.45 (1.99-3.02) | 6.31 (5.13-7.77) | 18.99 (15.40-23.40) | 33.98 (27.20-42.22) | 59.96 (47.45-75.78) | 64.62 (49.63-84.13) | 90.42 (67.70-120.75) | 121.48 (87.03-169.56) | 171.36 (115.54-254.15) | 187.60 (128.99-272.84) | <0.001 |
| Normonatremia patients on admission | | | | | | | | | | | |  |
| N，n | 64261 | 37210 | 13561 | 4743 | 2007 | 1024 | 546 | 297 | 159 | 69 | 89 |  |
| Mortality，n (%) | 101 (0.2) | 138 (0.4) | 152 (1.1) | 178 (3.8) | 152 (7.6) | 127 (12.4) | 87 (15.9) | 60 (20.2) | 34 (21.4) | 24 (34.9) | 34 (38.2) | <0.001 |
| Model 1^a^ | 1.00 | 2.20 (1.70-2.84) | 6.04 (4.69-7.79) | 19.40 (15.05-25.01) | 41.08 (31.42-53.69) | 72.33 (54.22-96.49) | 96.45 (69.10-134.62) | 130.78 (89.68-190.71) | 154.37 (97.27-244.98) | 267.97 (152.30-471.50) | 349.25 (201.89-604.16) | <0.001 |
| Model 2^b^ | 1.00 | 2.37 (1.83-3.07) | 6.81 (5.28-8.78) | 21.98 (17.03-28.37) | 44.83 (34.26-58.68) | 75.54 (56.48-101.04) | 91.42 (65.49-127.61) | 117.10 (80.66-170.00) | 128.12 (79.03-207.70) | 197.49 (110.66-352.45) | 240.88 (136.95-423.58) | <0.001 |
| Dysnatremia patients on admission | | | | | | | | | | | |  |
| N，n | 1668 | 2669 | 2604 | 1704 | 1014 | 631 | 427 | 275 | 180 | 111 | 143 |  |
| Mortality，n (%) | 48 (2.9) | 85 (3.2) | 97 (3.7) | 126 (7.4) | 94 (9.3) | 102 (16.2) | 66 (15.5) | 61 (22.2) | 56 (31.1) | 38 (34.2) | 51 (35.7) | <0.001 |
| Model 1^a^ | 1.00 | 1.27 (0.89-1.82) | 1.66 (1.15-2.38) | 3.67 (2.56-5.27) | 4.78 (3.27-6.99) | 8.88 (6.02-13.11_ | 8.64 (5.65-13.20) | 12.99 (8.24-20.47) | 21.68 (13.36-35.18) | 30.60 (18.12-51.70) | 31.16 (18.62-52.15) | <0.001 |
| Model 2^b^ | 1.00 | 1.23 (0.85-1.76) | 1.50 (1.04-2.16) | 3.28 (2.28-4.72) | 4.27 (2.91-6.26) | 7.11 (4.80-10.55) | 6.26 (4.04-9.70) | 9.57 (6.00-15.28) | 14.48 (8.75-23.96) | 19.35 (11.17-33.52) | 17.91 (10.41-30.80) | <0.001 |

Categorical variables are presented as counts and percentages. Cochran-Armitage test were used to examine trends among categorical variables and odds ratios.

a: Adjusted with age, sex, length of hospital stays, and Charlson Comorbidities Index.

b: Adjusted with age, sex, length of hospital stays, myocardial infarction, chronic pulmonary disease, moderate or severe liver disease, moderate or severe renal disease, metastatic solid tumor, serum sodium level on admission and average serum sodium level during hospitalization.

**Table S6.** The association between sodium fluctuation level (cutoff = 10 mmol/L) and mortality based on generalized estimated equations.

|  | **Serum sodium fluctuation level** | | | |  |
| --- | --- | --- | --- | --- | --- |
|  | 0-10.0 mmol/L | 10.0-20.0 mmol/L | 20.0-30.0 mmol/L | > 30.0 mmol/L | *P trend* |
| General hospitalized patients | | | | | |
| N，n | 124877 | 8994 | 1379 | 232 |  |
| Mortality，n (%) | 730 (0.6) | 766 (8.5) | 331 (24.0) | 85 (36.6) | <0.001 |
| Model 1^a^ | 1.00 | 13.03 (11.63-14.60) | 44.39 (37.59-52.41) | 90.90 (66.35-124.53) | <0.001 |
| Model 2^b^ | 1.00 | 12.99 (11.54-14.62) | 36.26 (30.43-43.21) | 62.61 (44.80-87.50) | <0.001 |
| Normonatremia patients on admission | | | | | |
| N，n | 117262 | 6042 | 663 | 89 |  |
| Mortality，n (%) | 447 (0.4) | 461 (7.6) | 146 (22.0) | 34 (38.2) | <0.001 |
| Model 1^a^ | 1.00 | 17.11 (14.86-19.70) | 57.40 (45.44-72.50) | 132.00 (80.94-215.27) | <0.001 |
| Model 2^b^ | 1.00 | 17.49 (15.10-20.25) | 45.33 (35.37-58.09) | 81.80 (48.05-139.23) | <0.001 |
| Dysnatremia patients on admission | | | | | |
| N，n | 7615 | 2952 | 716 | 143 |  |
| Mortality，n (%) | 283 (3.7) | 305 (10.3) | 185 (25.8) | 51 (35.7) | <0.001 |
| Model 1^a^ | 1.00 | 3.45 (2.86-4.15) | 10.70 (8.42-13.59) | 19.21 (12.65-29.18) | <0.001 |
| Model 2^b^ | 1.00 | 3.05 (2.53-3.68) | 7.54 (5.85-9.82) | 11.09 (7.12-17.27) | <0.001 |

Categorical variables are presented as counts and percentages. Results of models are presented in Odds Ratio and 95% Confidence Interval. Cochran-Armitage test was used to examine trends among categorical variables and odds ratios.

a: Adjusted with age, sex, length of hospital stays, and Charlson Comorbidities Index.

b: Adjusted with age, sex, length of hospital stays, myocardial infarction, chronic pulmonary disease, moderate or severe liver disease, moderate or severe renal disease, metastatic solid tumor, serum sodium level on admission and average serum sodium level during hospitalization.

**Table S7.** The association between minimum, maximum, fluctuation range of serum sodium level and in-hospital mortality based on generalized estimated equations.

|  | **Minimum serum sodium level, decrease per 1mmol/L** | ***P*** | **Maximum serum sodium level, increase per 1 mmol/L** | ***P*** | **Sodium fluctuation level during hospitalization, increase per 1 mmol/L** | ***P*** |
| --- | --- | --- | --- | --- | --- | --- |
| General hospitalized patients | | | | | | |
| Model 1^a^ | 1.39 (1.37-1.42) | <0.001 | 1.35 (11.33-1.38) | <0.001 | 1.20 (1.19-1.21) | <0.001 |
| Model 2^b^ | 1.15 (1.14-1.1.6) | <0.001 | 1.21 (1.20-1.23) | <0.001 | 1.21 (1.20-1.22) | <0.001 |
| Normonatremia patients on admission | | | | | | |
| Model 1^a^ | 1.48 (1.44-1.51) | <0.001 | 1.42 (1.38-1.45) | <0.001 | 1.24 (1.23-1.26) | <0.001 |
| Model 2^b^ | 1.20 (1.18-1.22) | <0.001 | 1.27 (1.25-1.30) | <0.001 | 1.25 (1.23-1.26) | <0.001 |
| Dysnatremia patients on admission | | | | | | |
| Model 1^a^ | 1.17 (1.15-1.20) | <0.001 | 1.18 (1.15-1.21) | <0.001 | 1.10 (1.09-1.11) | <0.001 |
| Model 2^b^ | 0.99 (0.97-1.01) | 0.216 | 1.12 (1.11-1.13) | <0.001 | 1.12 (1.11-1.14) | <0.001 |

Categorical variables are presented as counts and percentages. Results of models are presented in Odds Ratio and 95% Confidence Interval.

Abbreviation: AMA: against medical advice.

a: Adjusted with age, sex, length of hospital stays, myocardial infarction, chronic pulmonary disease, moderate or severe liver disease, moderate or severe renal disease, and metastatic solid tumor.

b: Adjusted with age, sex, length of hospital stays, and Charlson Comorbidities Index.

**Table S8.** The association between minimum, maximum, fluctuation range of serum sodium level and in-hospital mortality in non-AMA discharged patients based on generalized estimated equations.

|  | **Minimum serum sodium level, decrease per 1mmol/L** | ***P*** | **Maximum serum sodium level, increase per 1 mmol/L** | ***P*** | **Sodium fluctuation level during hospitalization, increase per 1 mmol/L** | ***P*** |
| --- | --- | --- | --- | --- | --- | --- |
| General hospitalized patients | | | | | | |
| Model 1^a^ | 1.40 (1.38-1.42) | <0.001 | 1.36 (1.34-1.39) | <0.001 | 1.21 (1.20-1.22) | <0.001 |
| Model 2^b^ | 1.15 (1.14-1.17) | <0.001 | 1.22 (1.21-1.24) | <0.001 | 1.22 (1.20-1.23) | <0.001 |
| Normonatremia patients on admission | | | | | | |
| Model 1^a^ | 1.49 (1.45-1.52) | <0.001 | 1.43 (1.40-1.47) | <0.001 | 1.25 (1.23-1.26) | <0.001 |
| Model 2^b^ | 1.20 (1.18-1.22) | <0.001 | 1.28 (1.26-1.31) | <0.001 | 1.25 (1.24-1.27) | <0.001 |
| Dysnatremia patients on admission | | | | | | |
| Model 1^a^ | 1.18 (1.15-1.21) | <0.001 | 1.18 (1.16-1.21) | <0.001 | 1.10 (1.09-1.12) | <0.001 |
| Model 2^b^ | 0.99 (0.97-1.01) | 0.205 | 1.12 (1.11-1.14) | <0.001 | 1.13 (1.11-1.14) | <0.001 |

Categorical variables are presented as counts and percentages. Results of models are presented in Odds Ratio and 95% Confidence Interval.

Abbreviation: AMA: against medical advice.

a: Adjusted with age, sex, length of hospital stays, myocardial infarction, chronic pulmonary disease, moderate or severe liver disease, moderate or severe renal disease, and metastatic solid tumor.

b: Adjusted with age, sex, length of hospital stays, and Charlson Comorbidities Index.

**Table S9.** In-hospital mortality of patients divided by cutoffs of sodium fluctuation level during hospitalization recognized by receiver operator characteristic curves in general hospitalized patients.

|  | **General hospitalized patients** | | | **Normonatremia patients on admission** | | | **Dysnatremia patients on admission** | | |
| --- | --- | --- | --- | --- | --- | --- | --- | --- | --- |
|  | < 7.5 mmol/L | > 7.5 mmol/L | *P* | < 7.5 mmol/L | > 7.5 mmol/L | *P* | < 7.5 mmol/L | > 7.5 mmol/L | *P* |
| N | 112905 | 22577 |  | 107634 | 16422 |  | 5271 | 6155 |  |
| In-hospital mortality, n (%) | 265 (0.2) | 1147 (5.1) | <0.001 | 143 (0.1) | 642 (3.9) | <0.001 | 122 (2.3) | 505 (8.2) | <0.001 |

Categorical variables are presented as counts and percentages. Results of models are presented in Odds Ratio and 95% Confidence Interval.

**Table S10.** In-hospital mortality of non-AMA discharged patients divided by cutoffs of sodium fluctuation level during hospitalization recognized by receiver operator characteristic curves in non-AMA discharged patients.

|  | **General hospitalized patients** | | | **Normonatremia patients on admission** | | | **Dysnatremia patients on admission** | | |
| --- | --- | --- | --- | --- | --- | --- | --- | --- | --- |
|  | < 7.5 mmol/L | > 7.5 mmol/L | *P* | < 7.5 mmol/L | > 7.5 mmol/L | *P* | < 7.5 mmol/L | > 7.5 mmol/L | *P* |
| N | 112905 | 22577 |  | 107634 | 16422 |  | 5271 | 6155 |  |
| In-hospital mortality, n (%) | 265 (0.2) | 1147 (5.2) | <0.001 | 143 (0.1) | 642 (3.9) | <0.001 | 165 (3.1) | 659 (10.7) | <0.001 |

Categorical variables are presented as counts and percentages. Results of models are presented in Odds Ratio and 95% Confidence Interval.

Abbreviation: AMA: against medical advice.

**Table S11.** Brier score of minimum serum sodium level, maximum serum sodium level, and sodium fluctuation level during hospitalization in predicting mortality.

|  | **Minimum serum sodium level** | **Maximum serum sodium level** | **Sodium fluctuation level** |
| --- | --- | --- | --- |
| Brier score | | | |
| General hospitalized patients | 0.014 | 0.013 | 0.013 |
| Normonatremia patients on admission | 0.009 | 0.008 | 0.008 |
| Dysnatremia patients on admission | 0.067 | 0.063 | 0.063 |

**Table S12.** Brier score of minimum serum sodium level, maximum serum sodium level, and sodium fluctuation level during hospitalization in predicting in-hospital mortality.

|  | **Minimum serum sodium level** | **Maximum serum sodium level** | **Sodium fluctuation level** |
| --- | --- | --- | --- |
| Brier score | | | |
| General hospitalized patients | 0.010 | 0.010 | 0.010 |
| Normonatremia patients on admission | 0.006 | 0.006 | 0.006 |
| Dysnatremia patients on admission | 0.052 | 0.049 | 0.049 |

**Table S13.** Brier score of minimum serum sodium level, maximum serum sodium level, and sodium fluctuation level during hospitalization in predicting mortality in non-AMA discharged patients.

|  | **Minimum serum sodium level** | **Maximum serum sodium level** | **Sodium fluctuation level** |
| --- | --- | --- | --- |
| Brier score | | | |
| General hospitalized patients | 0.010 | 0.010 | 0.010 |
| Normonatremia patients on admission | 0.006 | 0.006 | 0.006 |
| Dysnatremia patients on admission | 0.053 | 0.050 | 0.050 |

Abbreviation: AMA: against medical advice.
